# Study on the cooling effect of the parallel perforated ventilation subgrade in permafrost regions based on the numerical model

**DOI:** 10.1371/journal.pone.0317916

**Published:** 2025-01-30

**Authors:** Xiaolan Liu, Chuanwei Fu, Xinglei Cheng

**Affiliations:** Tianjin Key Laboratory of Soft Soil Characteristics and Engineering Environment, Tianjin Chengjian University, Tianjin, China; Shandong University of Technology, CHINA

## Abstract

The runway in permafrost regions has remarkable temperature sensitivity. Therefore, this paper puts forward to the parallel perforated ventilation subgrade. The reliability and validity of the finite element model of the runway with the parallel perforated ventilation subgrade are verified by comparing with the previous studies. And the cooling effect of the parallel perforated ventilation subgrade is analyzed. Results show that the parallel perforated ventilation subgrade has a significant cooling effect on the pavement and has little cooling effect in the natural ground. Compared with the non-ventilation subgrade, temperature time-history curves for the parallel perforated ventilation subgrade change periodically each year and are gradually lower with the growth of time. Temperature-depth curves for the parallel perforated ventilation subgrade change significantly at the insulation layer, the crushed rock layer, and the perforated ventilation. The air velocity and working time of the parallel perforated ventilation have no effect on the temperature of the surface layer, base layer, and subbase layer, have little effect on the temperature of the insulation layer, and have great effect on the temperature of the crushed rock layer and subgrade. This study provides scientific support for the design, construction, and maintenance of the runway in permafrost regions.

## Introduction

According to the "Air Silk Road" development goals of the 14th five-year plan of China, Civil Aviation Administration of China plans to build and rebuild 133 airports in permafrost regions in the next few years, and vigorously promotes the process of airport construction in permafrost regions. However, the physical and mechanical properties of soil in permafrost regions change significantly with the temperature, which can easily cause frost heaving, thawing settlement, and crack of the runway. The above-mentioned diseases of the runway seriously affect the aircraft safety of take-off and landing. Therefore, ensuring the temperature stability of the subgrade is an urgent problem to be solved for airport engineering in permafrost regions, which is helpful to reduce the diseases of the runway and keep the operation of the aircraft safe.

Ventilation has the advantage of effectively blocking the downward transfer of the solar radiation heat absorbed by the pavement, reducing the temperature of the permafrost soil at the bottom of the pavement by means of forced convection heat transfer, and maintaining the stability of the temperature field of the subgrade. Hence, scholars at home and abroad have carried out relevant research on the engineering application of ventilation in permafrost regions [1,2]. Moreover, domestic and foreign scholars put forward many kinds of ventilation subgrade treatment techniques in permafrost regions, such as common ventilation, perforated ventilation, ventilation with self-windward vent, ventilation-insulation material combination, and ventilation-crushed rock combination [3–5]. Niu et al. [6–9] analyzed the cooling effect of the common ventilation in railway engineering by means of indoor tests, field measurements and finite element analysis. And they found that the common ventilation raised the upper limit of the permafrost at the bottom of the subgrade in railway engineering. Ma et al. [10] discussed the cooling effect of the common ventilation in the Qinghai-Tibet Railway subgrades, and they found the cooling mechanism instability of the common ventilation subgrade. Yang et al. [11] studied the temperature characteristics of the common ventilation subgrade based on the experimental data in the Qinghai-Tibet Railway. And they analyzed the effectiveness of the common ventilation subgrade in protecting the permafrost based on the heat turnover at the bottom of the subgrade within 1 m, the slope position and the upper limit position of the permafrost. Li et al. [12] studied the influence of mean annual ground temperature and ice content on the cooling effect of the common ventilation subgrade based on the measured data. Zhu et al. [13] conducted the on-site monitoring test and found that the heat absorption intensity in the warm season was 2.4 times the heat release intensity in the cold season for the common ventilation subgrade. Meanwhile, they also found that the whole common ventilation subgrade was in a state of slow heat absorption. Hu et al. [14,15] improved the common ventilation into the perforated ventilation, and applied the method of numerical simulation to prove that the perforated ventilation could effectively reduce the temperature of the subgrade soil of the Qinghai-Tibet Railway. Liu et al. [16,17] conducted indoor tests on the cooling effect of the perforated ventilation, and they confirmed that the cooling effect of the perforated ventilation was better than that of the common ventilation. Meanwhile, they found that the perforated ventilation would produce a warming effect during the positive temperature period, and they suggested closing the ventilation during the positive temperature period. Zhang et al. [18] found that the perforated ventilation was conducive to the evaporation of water in the subgrade during the warm season and could help cool down the subgrade through laboratory tests. Sun et al. [19] studied the cooling effect of horizontal buried perforated ventilation on the surface of crushed rock subgrade based on the laboratory test. And they found that horizontal buried perforated ventilation is beneficial for the thermal stability of subgrade in high temperature permafrost regions. Su et al. [20] found that the cooling effect of the perforated ventilation was better than that of the common ventilation based on the short-term monitoring results of Qinghai-Tibet railway. Yu et al. [21–23] incorporated an automatic temperature control system into the ventilation, which could automatically open and close when the external temperature reached the set temperature. This measure could fully utilize the natural cold energy and effectively isolate the external hot air, achieving the purpose of maximum cooling of the subgrade. Meanwhile, they found that the cooling rate of the ventilation with self-windward vent was greater than that of the common ventilation. Li et al. [24] compared the cooling effect of the ventilation with self-windward vent and the common ventilation by numerical simulation and found that the ventilation with self-windward vent had the better cooling rate and cooling effect. Wu et al. [25] compared the cooling effect of the common ventilation subgrade, the crushed rock subgrade, and the insulation material subgrade. And they found that the common ventilation subgrade and the crushed rock subgrade had a good effect on raising the upper limit of the permafrost. Hou et al. [26] analyzed the temperature data of the ventilation-crushed rock combination subgrade based on the field test. And they found that the thickness of the crushed rock could affect the sunny- shady slope effect and improve the cooling effect.

In summary, most of the existing research focuses on the cooling effect of common ventilation, perforated ventilation, ventilation with self-windward vent, ventilation-insulation material combination, and ventilation-crushed rock combination subgrade in railway engineering and highway engineering. And some of the existing research also studies the upper limit of the permafrost, the temperature characteristics, the heat absorption intensity, and the sunny-shady slope effect of the ventilation subgrade in railway engineering and highway engineering. But few studies focus on the influence of ventilation on the cooling effect of subgrade in runway engineering. In fact, the pavement width, load-bearing capacity, and flatness of the runway in the airport engineering have higher requirements than those in railway and highway engineering. Moreover, the increase of pavement width leads to the heat absorption effect of the pavement increasing several times and causing greater disturbance to the underlying permafrost. The aircraft load acting on the runway is much larger than the train load acting on the railway and the vehicle load acting on the highway. In addition, the runway cannot be higher than the ground level, which results in no slope for the runway. And within a range around the runway, there should be no protrusions that could affect the safety of aircraft operations. All the above peculiarities for runway lead to the stability of temperature field in airport engineering is more difficult to control than that of highway and railway engineering. Hence, the ventilation subgrade for highways and railways in permafrost regions cannot be applied to runways in airport engineering and can only be used as a reference. Therefore, this paper proposes a new type of ventilation, which is named as the parallel perforated ventilation. Moreover, the combination of insulation layer, crushed rock layer, and parallel perforated ventilation is applied for the runway in permafrost regions. Finally, the cooling effect of the combination of insulation layer, crushed rock layer, and parallel perforated ventilation is analyzed based on temperature time-history curves and temperature-depth curves. The research provides scientific basis and technical support for the design and construction, operation and maintenance, and safety management of the runway in permafrost regions.

## Theory

### Three-dimensional unsteady heat transfer governing equation

With the change of the external temperature, the permafrost soil freezes when the temperature is below 0°C and thaws when the temperature is above 0°C. The phenomenon of freeze-thaw cycle results in the mutual transformation between solid phases and liquid phases of water in permafrost regions. Considering the water phase change in permafrost regions, it is necessary to take the phase change factor into establishing the three-dimensional unsteady heat transfer governing equation [27,28].

Freezing zones:

$$C_{f}\frac{\partial T_{f}}{\partial t}=\frac{\partial }{\partial x}(\lambda_{f}\frac{\partial T_{f}}{\partial x})+\frac{\partial }{\partial y}(\lambda_{f}\frac{\partial T_{f}}{\partial y})+\frac{\partial }{\partial z}(\lambda_{f}\frac{\partial T_{f}}{\partial z})+q_{s}$$ (1)

Thawing zones:

$$C_{u}\frac{\partial T_{u}}{\partial t}=\frac{\partial }{\partial x}(\lambda_{u}\frac{\partial T_{u}}{\partial x})+\frac{\partial }{\partial y}(\lambda_{u}\frac{\partial T_{u}}{\partial y})+\frac{\partial }{\partial z}(\lambda_{u}\frac{\partial T_{u}}{\partial z})+q_{l}$$ (2)

Eqs (1) and (2) have a continuous temperature and meet energy conservation at the phase transition interface, as shown in Eqs (3) and (4) [27,28].


$$T_{f}(s(t),t)=T_{u}(s(t),t)=T_{m}$$ (3)

$$\lambda_{f}\frac{\partial T_{f}}{\partial n}-\lambda_{u}\frac{\partial T_{u}}{\partial n}=L\gamma_{d}\left(W-W_{u}\right)\frac{ds(t)}{dt}$$ (4)

Here, *x*, *y*, and *z* are the rectangular coordinates. *t* is the time. *C*_*f*_ and *C*_*u*_ are the heat capacity of soil in the freezing and thawing zones, respectively. *T*_*f*_ and *T*_*u*_ are the temperature of soil in the freezing and thawing zones, respectively. *λ*_*f*_ and *λ*_*u*_ are the thermal conductivity of soil in the freezing and thawing zones, respectively. *q*_*s*_ and *q*_*l*_ are the heat source intensity of soil in the freezing and thawing zones, respectively. *T*_*m*_ is the soil temperature of phase transition interface. *W* is the deformation energy of soil. *W*_*u*_ is the phase transition energy of soil in the thawing zone. *L* is the phase change latent heat of soil. *γ*_*d*_ is the dry density of soil.

### Governing equations of gas flowing in porous media and ventilation

The crushed rock layer is a porous medium. The characteristics of the pore shape, size and direction are not arranged regularly and complex structure. The flow gas in the pore changes its shape with the change of the pore. Hence, the porous medium is assumed to be an ideal continuous medium superimposed by solid and fluid, and the solid pores are filled with fluid. The averaged momentum equation of the porous media is obtained by considering the inertia term and the non-slip condition of porous wall medium, as shown in Eq (5) [27,28].


$$\rho_{f}(V^{\prime }\cdot \nabla V^{\prime })=-\nabla p+\mu_{f}\nabla^{2}V^{\prime }-\frac{\mu_{f}\phi }{K}V^{\prime }-\rho_{f}\frac{F\phi^{2}}{\sqrt{K}}|V^{\prime }|V^{\prime }$$ (5)

Here, *ρ*_*f*_ is the density of fluid. *V’* is the average speed of fluid. *μ*_*f*_ is the viscosity coefficient of fluid. *Φ* is the porosity of porous medium. *K* is the permeability. *F* is the coefficient of inertia term.

In accordance with the theorem of Kozeny-Carmen, the permeability and the coefficient of inertia term in Eq (5) can be described as Eqs (6) and (7) [27,28].


$$K=\frac{\phi^{3}d_{p}^{2}}{150{\left(1-\phi \right)}^{2}}$$ (6)

$$F=\frac{1.75}{\sqrt{150}\phi^{\frac{3}{2}}}$$ (7)

It is assumed that the solids and fluids in a porous medium reach a local thermal equilibrium, which means that the mean solid temperature equals the mean fluid temperature. The energy equation can be expressed as Eq (8) [27,28].


$$\rho_{f}\frac{\partial h_{f}}{\partial t}+\rho_{f}\nabla (V^{\prime }h_{f})=\nabla [\frac{\left(\lambda_{m}+\lambda_{d}\right)}{c_{f}}\nabla h_{f}]$$ (8)

Here, *h*_*f*_ is the specific enthalpy of fluid. *λ*_*m*_ is the thermal conductivity when the flow of fluid is stagnant. *λ*_*d*_ is the thermal conductivity of thermal dispersion. *c*_*f*_ is the specific heat capacity of fluid.

When *Φ* is equal to 1, Eqs (5)–(8) are the governing equation of gas flowing in the ventilation.

### Boundary conditions

The top of the surface layer is affected by external factors, including solar radiation, rain, snow and wind. Meanwhile, the top of the surface layer is also affected by internal factors, such as geological structure, material properties, water content, and so on. Hence, the following assumptions are made for the runway model. ① the pavement structure and subgrade are all incompressible and isotropic. ② it does not consider the water transfer in the structure layers and between the contact surfaces. ③ the pore of ventilation is even, not blocked, and not damaged in the whole service life. ④ the pore connectivity in the crushed rock layer can ensure the gas flow, and the gas is not compressible and does not occur chemical reaction. ⑤ the evaporative and heat dissipation are ignored in the calculation. Therefore, according to the boundary layer theory, the lower boundary layer with stable temperature variation is taken as the upper boundary temperature condition of the finite element model, as shown in Eq (9). The boundary condition at the bottom of the finite element model is assumed to be a constant heat flux considering the geothermal effect, as shown in Eq (10). In combination with the temperature boundary effect, the boundary conditions on both sides of the finite element model are adiabatic, as shown in Eq (11) [27,28].


$$T=T_{0}+\Delta T+a\;\sin(\frac{2\pi t}{8760}+b)+\frac{\alpha t}{8760\times 30}$$ (9)

$$\lambda \frac{\partial T}{\partial n}=0.03$$ (10)

$$\lambda \frac{\partial T}{\partial n}=0$$ (11)

Here, *T*_0_ is the mean annual ground temperature of the lower boundary layer, taken as the mean annual air temperature. Δ*T* is the temperature increment of the lower boundary layer and is set to 4.5°C for the pavement and 2.5°C for the natural surface. *a* is the variation amplitude of temperature and is set to 15°C for the pavement and 12°C for the natural surface. *b* is the initial phase and equals to zero at the initial set time of April 1 in the northeast of China. *α* is the increment in the global temperature over the next 30 years and is set to 1.5°C. *T* is the temperature. *λ* is the thermal conductivity. *n* is the normal direction.

## Numerical model

### Numerical model establishment

**Fig 1:**
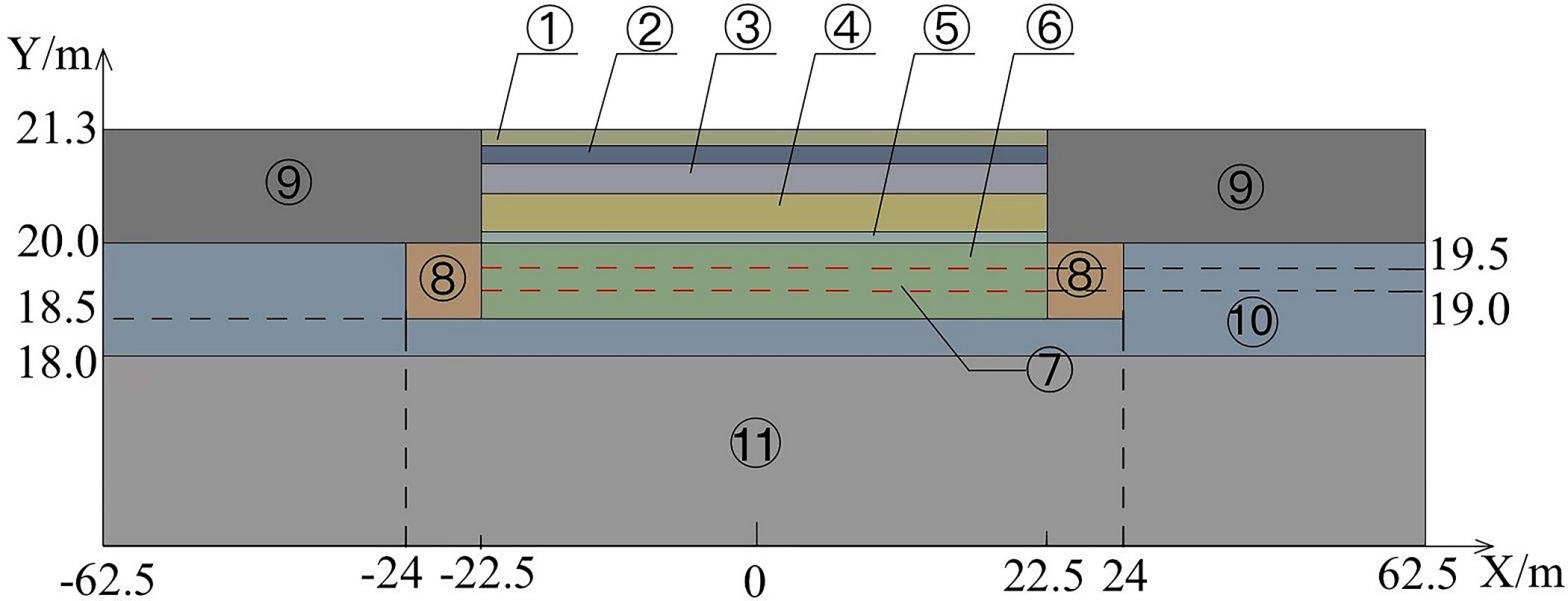
Three-dimensional model of the runway at front view (① the upper -surface layer of asphalt concrete, ② the under-surface layer of asphalt concrete, ③ base layer, ④ subbase layer, ⑤ insulation layer, ⑥ crushed rock layer, ⑦ circular perforated ventilation, ⑧ rectangular duct, ⑨ clay, ⑩ silty clay, ⑪ strongly weathered rock).

**Fig 2:**
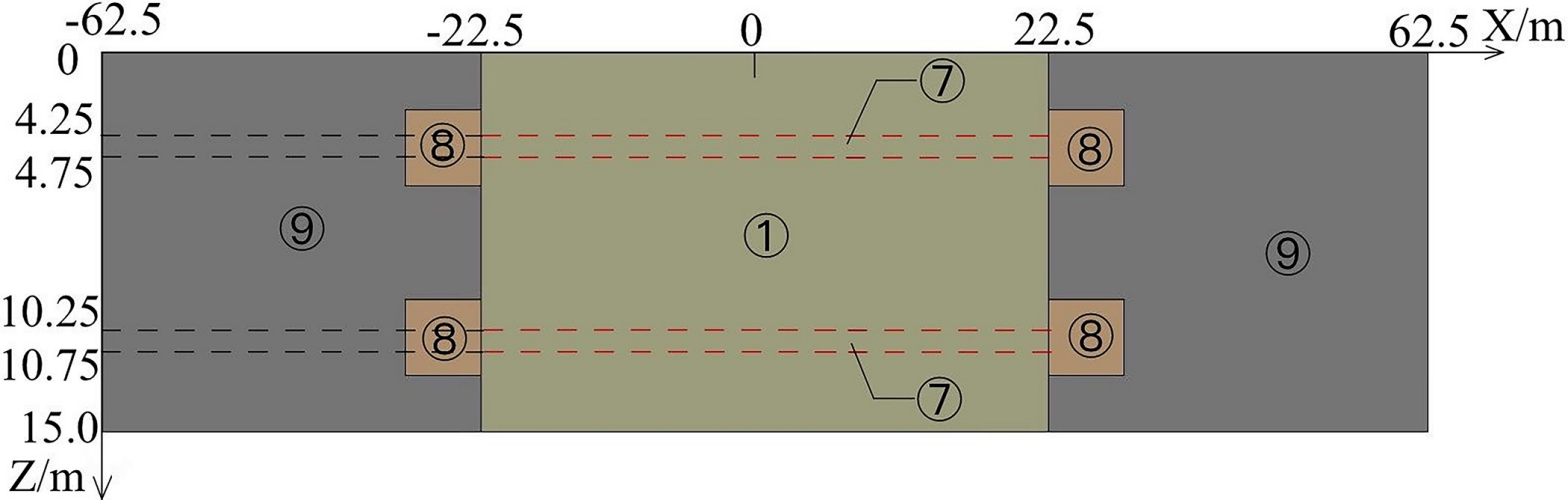
Three-dimensional model of the runway at vertical view (① the upper -surface layer of asphalt concrete, ② the under-surface layer of asphalt concrete, ③ base layer, ④ subbase layer, ⑤ insulation layer, ⑥ crushed rock layer, ⑦ circular perforated ventilation, ⑧ rectangular duct, ⑨ clay, ⑩ silty clay, ⑪ strongly weathered rock).

**Fig 3:**
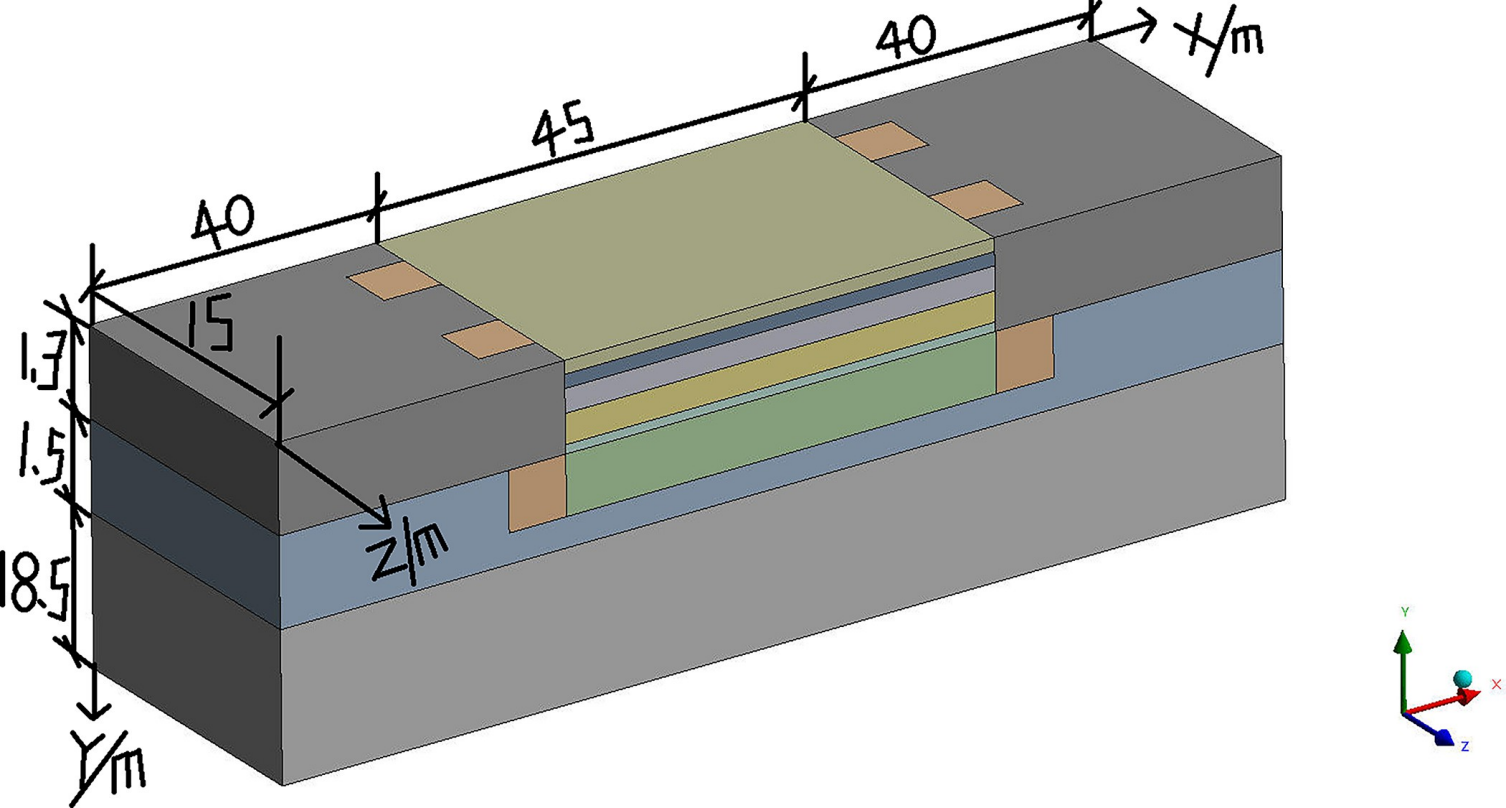
Three-dimensional model of the runway at three-dimensional view (① the upper -surface layer of asphalt concrete, ② the under-surface layer of asphalt concrete, ③ base layer, ④ subbase layer, ⑤ insulation layer, ⑥ crushed rock layer, ⑦ circular perforated ventilation, ⑧ rectangular duct, ⑨ clay, ⑩ silty clay, ⑪ strongly weathered rock).

**Fig 4:**
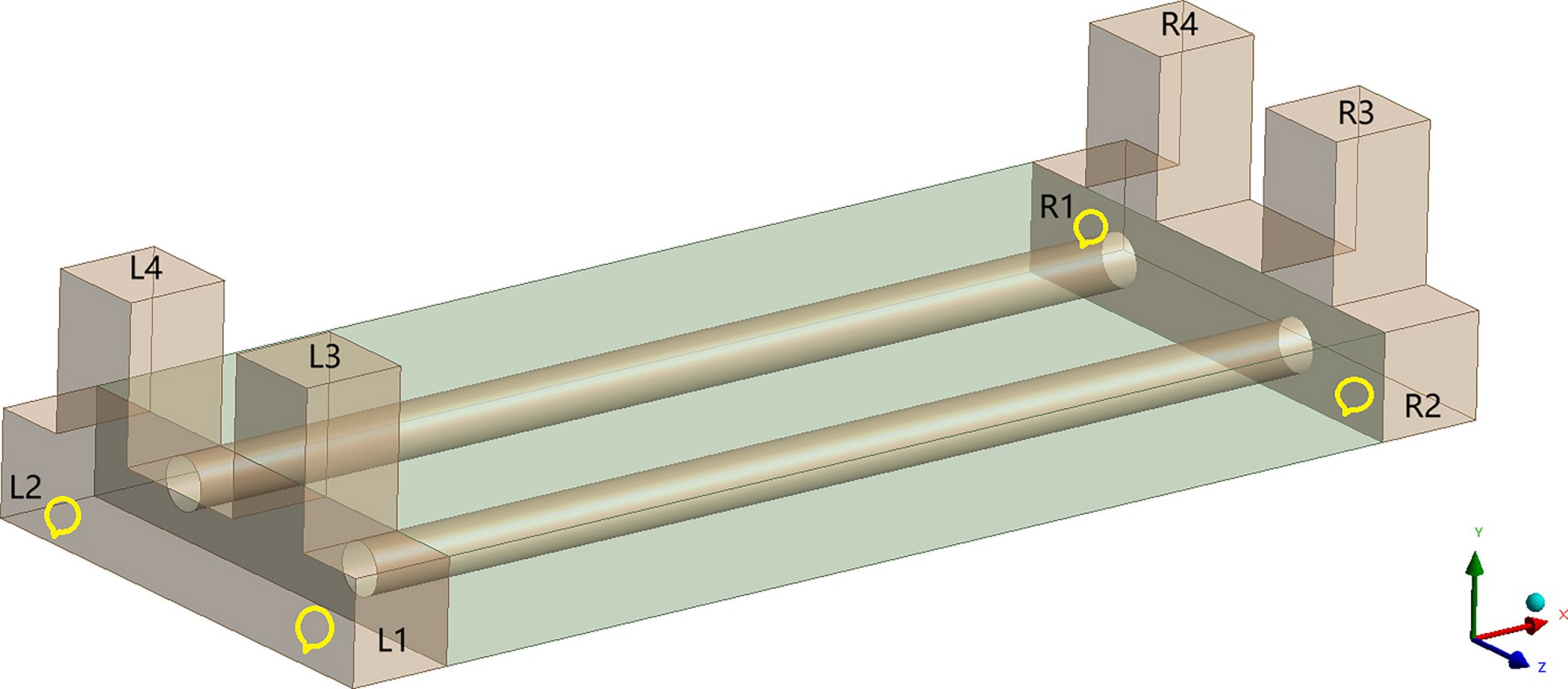
Three-dimensional model of parallel perforated ventilation.

The 4E level is in the middle-upper level of airfield area class and can accommodate the take-off and landing of most aircraft. And according to *Technical Standards for Airfield Area of Civil Airports*, the airfield area class of 4E level must meet that the pavement width is 45m [27]. And the runway width must accommodate that the distance between the outer wheels of the main landing gear is 9m-14m, and the aircraft wingspan is 52m-65m [27]. Meanwhile, considering the effect of the size and calculation time, the pavement of the runway is 15m length at the direction of Z and 45m width at the direction of X, and the subgrade of the runway is 18.5m height at the direction of Y. Both sides of the pavement extend outward 40m in the X direction to eliminate the influence of the boundary effect on the temperature field of the runway. According to *Specification for asphalt pavement design of civil airports*, the minimum thickness of surface layer and base layer shall not be less than 0.3m and the minimum thickness of the subbase layer is no less than 0.5m to meet the requirements of frost prevention when the airfield area indicators II is E [28]. Meanwhile, considering the relevant research results [29,30], the thickness of the surface layer is selected to be 0.3m, the thickness of the base layer is selected to be 0.4m, and the thickness of the subbase layer is selected to be 0.5m. Therefore, the runway with the parallel perforated ventilation subgrade is shown on Figs 1–3 (In Figs 1 and 2, 62.5m-22.5m = 40m, -62.5m-(-22.5m) = -40m, 22.5m-(-22.5m) = 45m.). The parallel perforated ventilation is made of circular perforated ventilation and rectangular duct, as shown in Fig 4. The direction of Z is the taxiing direction of the aircraft, and the initial 0m is at the starting point of the natural ground. The direction of X is perpendicular to the taxiing direction of the aircraft, and the initial 0m is in the center of the pavement. The direction of Y is the depth direction of the runway, and the initial 0m is at the bottom of the subgrade. The temperature boundary condition at the surface of the pavement and natural ground is expressed as Eq (9). The temperature boundary condition on the left-right and front-behind sides of the runway model is expressed as Eq (11). The temperature boundary condition at the bottom of the subgrade is expressed as Eq (10). As the thermal parameters of the soil are closely related to temperature, the thermal parameters of soil are shown in Table 1 considering ice-water phase transition and heat transfer [29,30]. As the thermal parameters of the pavement and crushed rock layer are very little affected by the temperature, the thermal parameters of the pavement and crushed rock layer are shown in Table 2 without the influence of the temperature [29,30]. In the finite element model, a pair of coupled surfaces, called “wall” and “wall-shadow”, are automatically generated after the mesh is imported. Since the “wall” surface is determined as the boundary condition with the “coupled” option, the finite element model of the runway automatically realizes the fluid and solid coupled heat transfer. Meanwhile, based on the governing equations of gas flowing in porous media and ventilation, the “interior” in the finite element model is applied to realize the fluid and solid coupled heat transfer and fluid penetration. The velocity and temperature of air at the inlet is 5m/s and −30°C, respectively. The air velocity is normal to the boundary. The air velocity at the outlet is set as the pressure boundary with the turbulent intensity of 5% and the turbulent viscosity ratio of 10.

**Table 1:** The thermal parameters of the soil.

Type	Thickness/m	Thermal parameter	-20°C	-10°C	-5°C	-2°C	-1°C	-0.5°C	0°C	20°C
**Clay**	1.3	Density/(kg/m^3^)	1870	1870	1870	1870	1870	1870	1870	1870
		Thermal conductivity/ (J/(m·h·°C))	8640	8640	8640	8640	8640	8640	5544	5544
		Heat capacity/ (J/(kg·°C))	835	840	850	860	870	900	1070	1070
**Silty clay**	2	Density/(kg/m^3^)	1950	1950	1950	1950	1950	1950	1950	1950
		Thermal conductivity/ (J/(m·h·°C))	6500	6500	6500	6500	6500	6500	5400	5400
		Heat capacity/ (J/(kg·°C))	970	1050	1090	1115	1140	1210	1285	1285
**Strongly weathered rock**	18	Density/(kg/m^3^)	2150	2150	2150	2150	2150	2150	2150	2150
		Thermal conductivity/ (J/(m·h·°C))	9000	9000	9000	9000	9000	9000	7250	7250
		Heat capacity/ (J/(kg·°C))	950	1060	1110	1140	1190	1250	1350	1350

**Table 2:** The thermal parameters of the pavement and crushed rock layer.

Number	Structure	Material	Thickness/m	Density/ (kg/m^3^)	Thermal conductivity/ (J/(m·h·°C))	Heat capacity/ (J/(kg·°C))
**①**	The upper-surface layer of asphalt concrete	AC-13C	0.15	2300	4140	1670
**②**	The under-surface layer of asphalt concrete	AC-20C	0.15	2320	4320	1670
**③**	Base layer	Cement stabilized crushed rock	0.4	2200	3960	960
**④**	Subbase layer	Graded sand and stone	0.5	2100	3240	2000
**⑤**	Insulation layer	Expanded polystyrene board (EPS)	0.1	40	108	1400
**⑥**	Crushed rock layer	Particle size of 6–8 cm	1.5	1490	1426	839

### The working mode of the parallel perforated ventilation

As shown in Fig 4, 1# and 2# rectangular ducts are made of steel net on the side near the pavement, which is convenient for air convection and heat transfer in the circular perforated ventilation, rectangular duct and crushed rock layer. L1, L2, R1, and R2 are the inlets of air, which place a blowing machine to blow cold air into the rectangular duct, respectively. L3, L4, R3, and R4 are outlets of air, which are used for discharging the air in the ventilation to the outside environment. L1, L2, R1, R2, L3, L4, R3, and R4 are equipped with closing devices to prevent outside heat from entering the runway during the warm season. The parallel perforated ventilation makes up for the lack of slop in the runway, promotes the cold air flowing in the crushed rock layer, enhances the convective heat transfer and reduces the temperature of subgrade remarkably. The working period of the parallel perforated ventilation is from December 22 to January 30 every year. The air velocity at the inlet is 5m/s, and the air temperature at the inlet is -30°C. The first working period is from December 22 to December 31, and the first working mode takes L1 as the air inlet and takes R1, R2, R3, R4 as the air outlet. The second working period is from January 1 to January 10, and the second working mode takes R1 as the air inlet and takes L1, L2, L3, L4 as the air outlet. The third working period is from January 11 to January 20, and the third working mode takes L2 as the air inlet and takes R1, R2, R3, R4 as the air outlet. The fourth working period is from January 21 to January 30, and the fourth working mode takes R2 as the air inlet and takes L1, L2, L3, L4 as the air outlet.

### Numerical model validation

**Fig 5:**
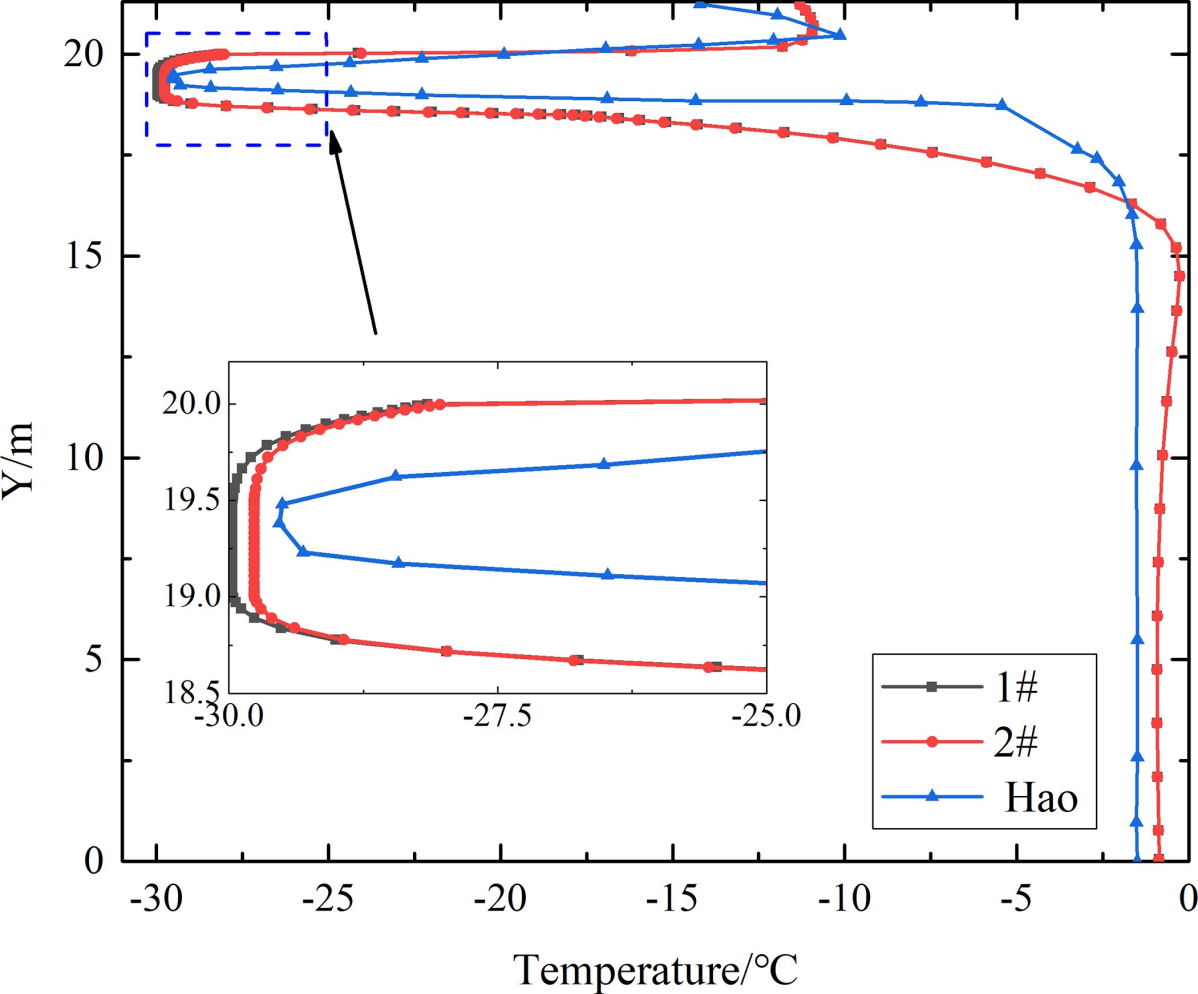
Temperature-depth curves at the middle of 1# and 2# perforated ventilation (the air velocity is 5m/s, and the time is January 5).

Due to the lack of monitoring data from the parallel perforated ventilation subgrade in permafrost regions, the reliability and validity of the finite element model of the runway with the parallel perforated ventilation subgrade are verified through comparison with the temperature-depth curves at different time of the experimental results in previous studies, as shown in Fig 5.

Fig 5 shows that the temperature rises first and then decreases with an increase of the depth at the part of pavement (Y = 20.0m-21.3m). The temperature drops precipitously at the top of the crushed rock layer (Y = 20.0m). The lowest temperature approaches -30°C at the top of the perforated ventilation (Y = 19.5m). The lowest temperature is kept in the perforated ventilation. The temperature gradually increases from the lower wall of the perforated ventilation (Y = 19.0m) to the bottom of the crushed rock layer (Y = 18.5m). The increasing rate of temperature gradually slows down within 0.5m at the bottom of the crushed rock layer (Y = 18.0m-18.5m). The temperature tends to a stable fluctuation value at 2.7m away from the bottom of the crushed rock layer (Y = 15.8m). In the study of Hao [29], the parameters of the soil, pavement and crushed rock layer are the same as Tables 1 and 2. The combination of insulation layer, crushed rock layer, and perforated ventilation is applied to cold down the temperature of subgrade. The velocity and temperature of air at the inlet of the perforated ventilation is 5m/s and −30°C, respectively. The working period is from January 1 to January 15. However, the structure of the parallel perforated ventilation in the paper is different from that of the perforated ventilation in the study of Hao [29], as shown in Fig 4. Compared to the perforated ventilation, the parallel perforated ventilation comprises two additional rectangular ducts and eight additional outlets. This structure is conducive to transmitting cold energy into the perforated ventilation, crushed rock layer, and subgrade during the cold season to lower the temperature of the permafrost and isolating external heat during the warm season to ensure the stability of the permafrost. Moreover, the working conditions of the parallel perforated ventilation are different from those of the perforated ventilation in the study of Hao [29], as shown in the part of “The working mode of the parallel perforated ventilation”. The above characteristics of the parallel perforated ventilation result in the cooling efficiency of the parallel perforated ventilation is higher than that of the perforated ventilation. Therefore, the shapes of the temperature-depth curves for the 1# and 2# perforated ventilation are almost the same, and both are similar to the study of Hao [29]. But the temperature at the top of the surface layer, in the perforated ventilation, at the bottom of the crushed rock layer, and at the bottom of the subgrade are not the same as the study of Hao [29].

The reason for the temperature-depth curve in Fig 5 is that the cold energy of air in the external environment is gradually consumed with the increase of the depth. And the temperature rises with the increase of the depth before reaching the insulation layer. But the insulation layer prevents the temperature of the pavement from passing down and prevents the cold air in the crushed rock layer from passing up. Hence, the cooling effect of the convective heat transfer in the crushed rock layer is remarkable, and the temperature dropping precipitously at the top of the crushed rock layer and reaching the lowest value in the perforated ventilation. Because the flow resistance of the cold air in the crushed rock layer is larger than that in the perforated ventilation, the temperature gradually increases from the lower wall of the ventilation to the bottom of the crushed rock layer. Meanwhile, the heat conduction in the silty clay layer and strongly weathered rock layer is dominant, and the convective heat transfer of cold air in the silty clay layer and strongly weathered rock layer is very weak. But the cooling effect of the convective heat transfer is much better than that of the heat conduction. Thereby, the temperature tends to a stable fluctuation value at 2.7m away from the bottom of the crushed rock layer.

## Results analysis

To explore the cooling effect of the parallel perforated ventilation subgrade in airport engineering, temperature time-history curves and temperature-depth curves for the non-ventilation subgrade and parallel perforated ventilation subgrade are compared for 30 years. And the influence of the air velocity and working time on the cooling effect of the parallel perforated ventilation subgrade in airport engineering are analyzed for 30 years.

### Temperature time-history curves for the non-ventilation subgrade and the parallel perforated ventilation subgrade

1. Temperature time-history curves in the center of the pavement
2. Temperature time-history curves in the center of the natural ground

**Fig 6:**
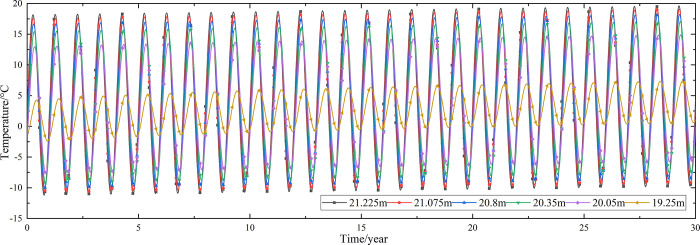
Temperature time-history curves at the depth of 19.25m-21.225m for the non-ventilation subgrade in the center of the pavement.

**Fig 7:**
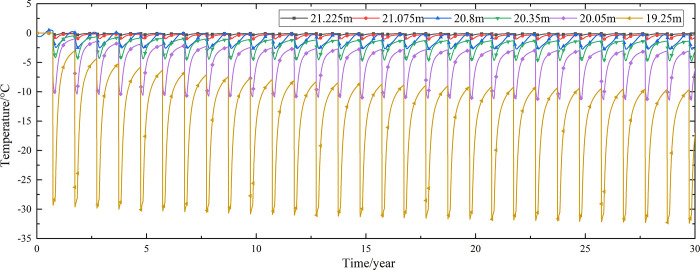
Temperature time-history curves at the depth of 19.25m-21.225m for the temperature difference between the non-ventilation subgrade and the parallel perforated ventilation subgrade in the center of the pavement.

Temperature time-history curves of the non-ventilation subgrade at the depth of 19.25m-21.225m are shown in Fig 6. The temperature difference between the non-ventilation subgrade and the parallel perforated ventilation subgrade at the depth of 19.25m-21.225m is shown in Fig 7. The middle of the upper-surface layer of asphalt concrete is at the depth of Y = 21.225m, the middle of the under-surface layer of asphalt concrete is at the depth of Y = 21.075m, the middle of the base layer is at the depth of Y = 20.8m, the middle of the subbase layer is at the depth of Y = 20.35m, the middle of the insulation layer is at the depth of Y = 20.05m, and the middle of the crushed rock layer is at the depth of Y = 19.25m.

Fig 6 shows that the temperature of the surface layer, base layer, subbase layer, insulation layer, and crushed rock layer change periodically with the ambient temperature every year. The temperature of the surface layer, base layer, subbase layer change significantly with the ambient temperature. The temperature of the insulation layer is less affected by the ambient environment compared with the temperature of the surface layer, base layer, and subbase layer. The temperature of the crushed rock layer is least affected by the ambient temperature. Fig 7 shows that the temperature difference between the non-ventilation subgrade and the parallel perforated ventilation subgrade decreases with the increasing of the distance away from the perforated ventilation. The temperature difference begins to increase when the perforated ventilation begins to work. The temperature difference reaches the maximum value when the perforated ventilation stops working, and then the temperature difference begins to decrease. The crushed rock layer is positive temperature only in spring and summer of the first year and is negative temperature in the whole year for the rest time. This phenomenon shows that the parallel perforated ventilation subgrade can effectively reduce the temperature of the insulation layer and crushed rock layer. The reason is that the temperature of the surface layer, base layer, and subbase layer are significantly affected by the ambient temperature and the ambient temperature is affected by the effect of global warming. The cooling effect of the parallel perforated ventilation subgrade is stronger than the effect of global warming, and the cooling effect of the parallel perforated ventilation subgrade is stronger and stronger with the increasing of time. Moreover, because the convection heat transfer and heat conduction all occur in the crushed rock layer, the cooling effect of the parallel perforated ventilation is most significant in the crushed rock layer. In addition, energy loss occurs when temperature is transferred to depth.

**Fig 8:**
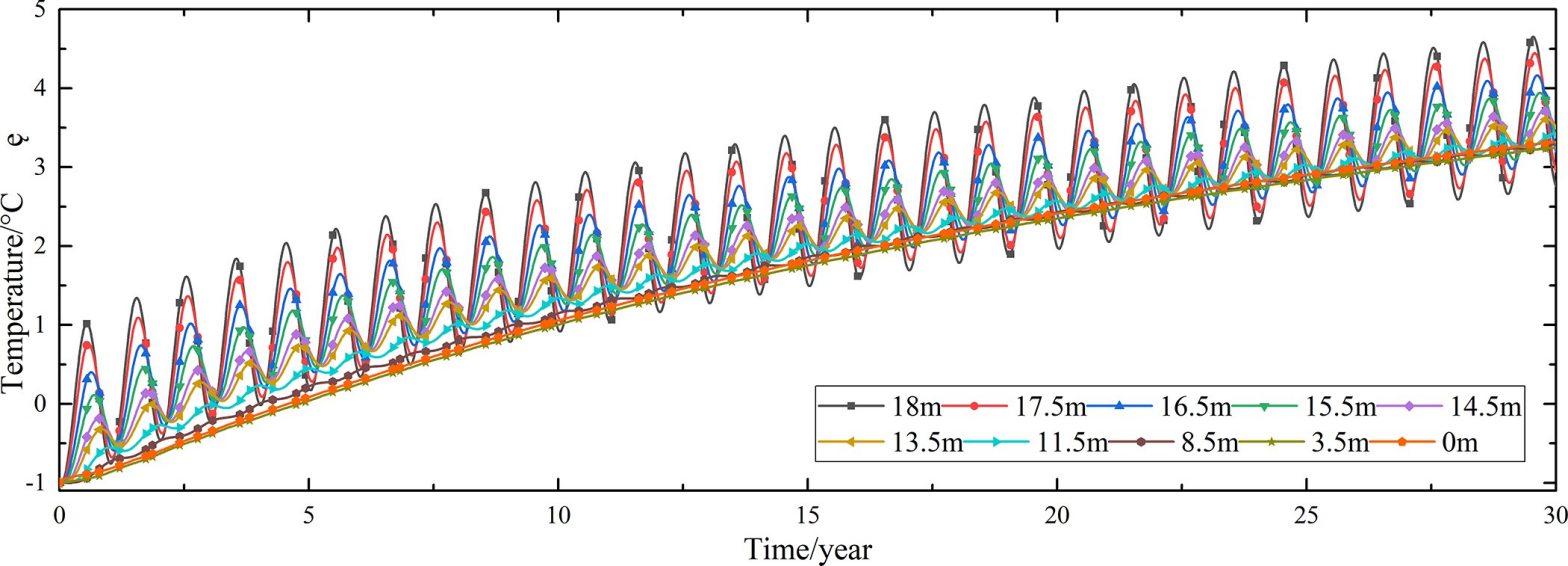
Temperature time-history curves at the depth of 0m-18m for the non-ventilation subgrade in the center of the pavement.

**Fig 9:**
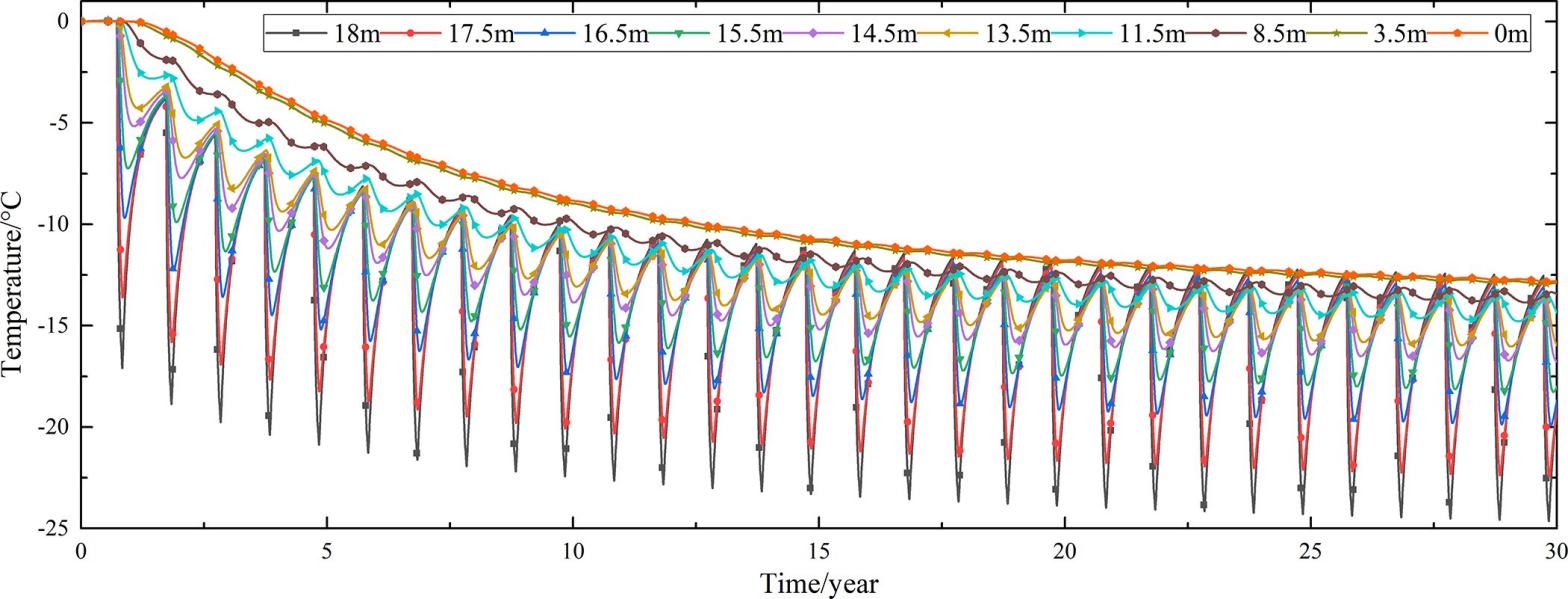
Temperature time-history curves at the depth of 0m-18m for the temperature difference between the non-ventilation subgrade and the parallel perforated ventilation subgrade in the center of the pavement.

Temperature time-history curves of the non-ventilation subgrade at the depth of 0m-18m are shown in Fig 8. The temperature difference between the non-ventilation subgrade and the parallel perforated ventilation subgrade at the depth of 0m-18m is shown in Fig 9. The 0.5m, 1.0m, 2.0m, 3.0m, 4.0m, 5.0m, 7.0m, 10.0m, 15.0m and 18.5m away from the bottom of the crushed rock layer are at the depth of Y = 18m, Y = 17.5m, Y = 16.5m, Y = 15.5m, Y = 14.5m, Y = 13.5m, Y = 11.5m, Y = 8.5m, Y = 3.5m, and Y = 0m, respectively.

Fig 8 shows that the temperature at the depth of Y = 18m, Y = 17.5m, Y = 16.5m, Y = 15.5m, Y = 14.5m, and Y = 13.5m change periodically with the ambient temperature. And the temperature at the depth of Y = 18m, Y = 17.5m, Y = 16.5m, Y = 15.5m, Y = 14.5m, and Y = 13.5m are negative only in the winter of the first two years and are positive the whole year for the rest of the time. The temperature at the depth of Y = 11.5m is less affected by the ambient environment. And the temperature at the depth of Y = 11.5m is negative only for the first four years and is positive the whole year for the rest of the time. The temperature at the depth of Y = 8.5m, Y = 3.5m, and Y = 0m are little affected by the ambient environment. And the temperature at the depth of Y = 8.5m, Y = 3.5m, and Y = 0m are negative only for the first five years and are positive the whole year for the rest of the time. Fig 9 shows that the temperature difference law is similar to that of Fig 7, and the decreasing amplitude is larger than that of Fig 7. The temperature at the depth of Y = 18m, Y = 17.5m, Y = 16.5m, Y = 15.5m, Y = 14.5m, Y = 13.5m and Y = 11.5m are positive only in the summer of the first year and are negative the whole year for the rest of the time. The temperature at the depth of Y = 8.5m is less affected by the ambient environment. The temperature at the depth of Y = 3.5m and Y = 0m are little affected by the ambient environment. And the temperature at the depth of Y = 8.5m, Y = 3.5m, and Y = 0m are negative in the whole year for the thirty years. This phenomenon shows that the parallel perforated ventilation subgrade can effectively reduce the temperature of the subgrade. The reason is that the cooling effect of the parallel perforated ventilation subgrade is stronger than the effect of global warming. And the cooling effect of the parallel perforated ventilation subgrade is stronger and stronger with the increasing of the time. Moreover, because the convection heat transfer and heat conduction all occur in the subgrade, the cooling effect of the parallel perforated ventilation subgrade is better in the subgrade than that in the pavement. In addition, energy loss occurs when temperature is transferred to depth.

**Fig 10:**
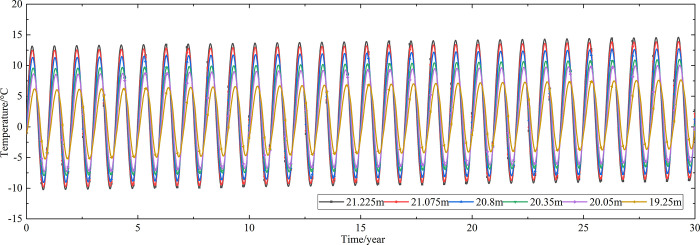
Temperature time-history curves at the depth of 19.25m-21.225m for the non-ventilation subgrade in the center of the natural ground.

**Fig 11:**
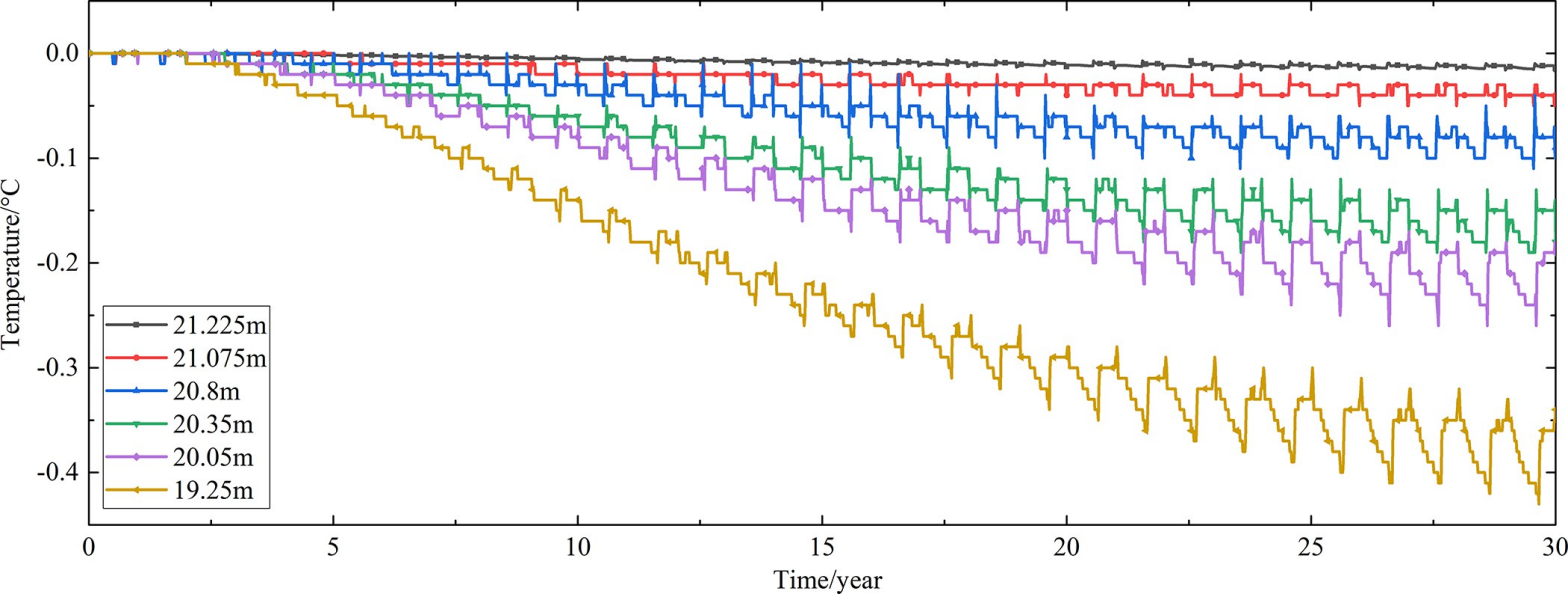
Temperature time-history curves at the depth of 19.25m-21.225m for the temperature difference between the non-ventilation subgrade and the parallel perforated ventilation subgrade in the center of the natural ground.

**Fig 12:**
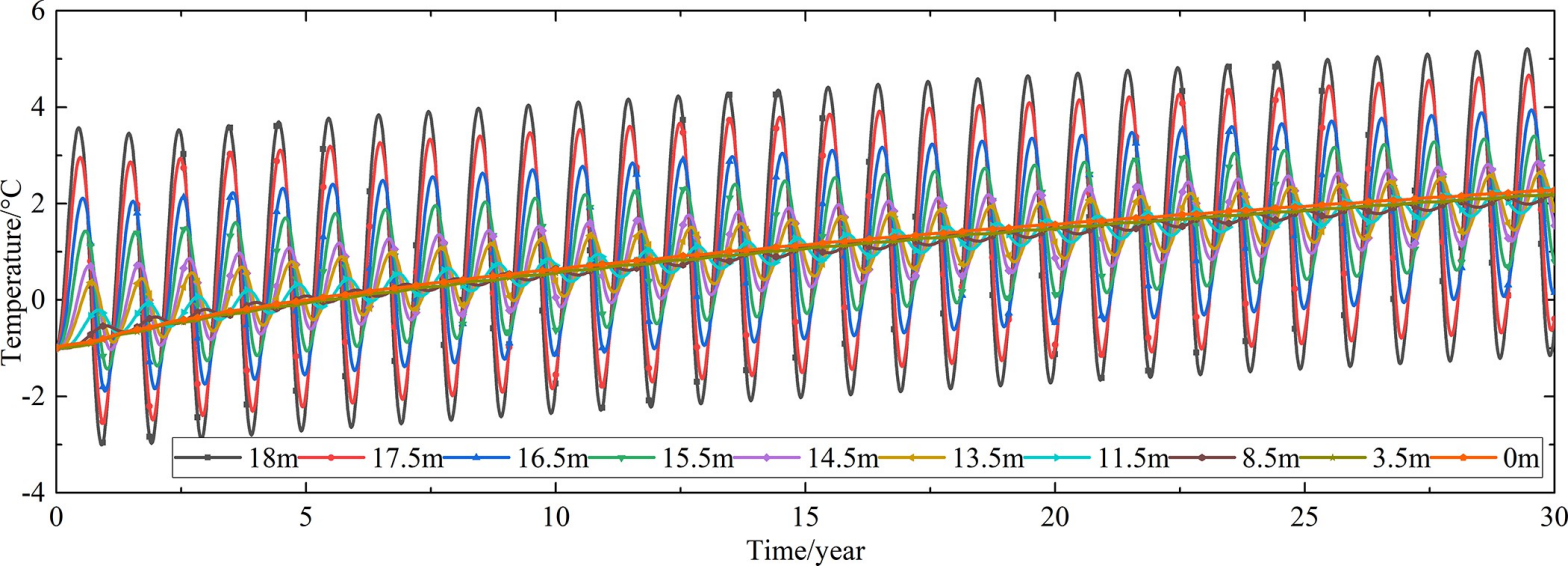
Temperature time-history curves at the depth of 0m-18m for the non-ventilation subgrade in the center of the natural ground.

**Fig 13:**
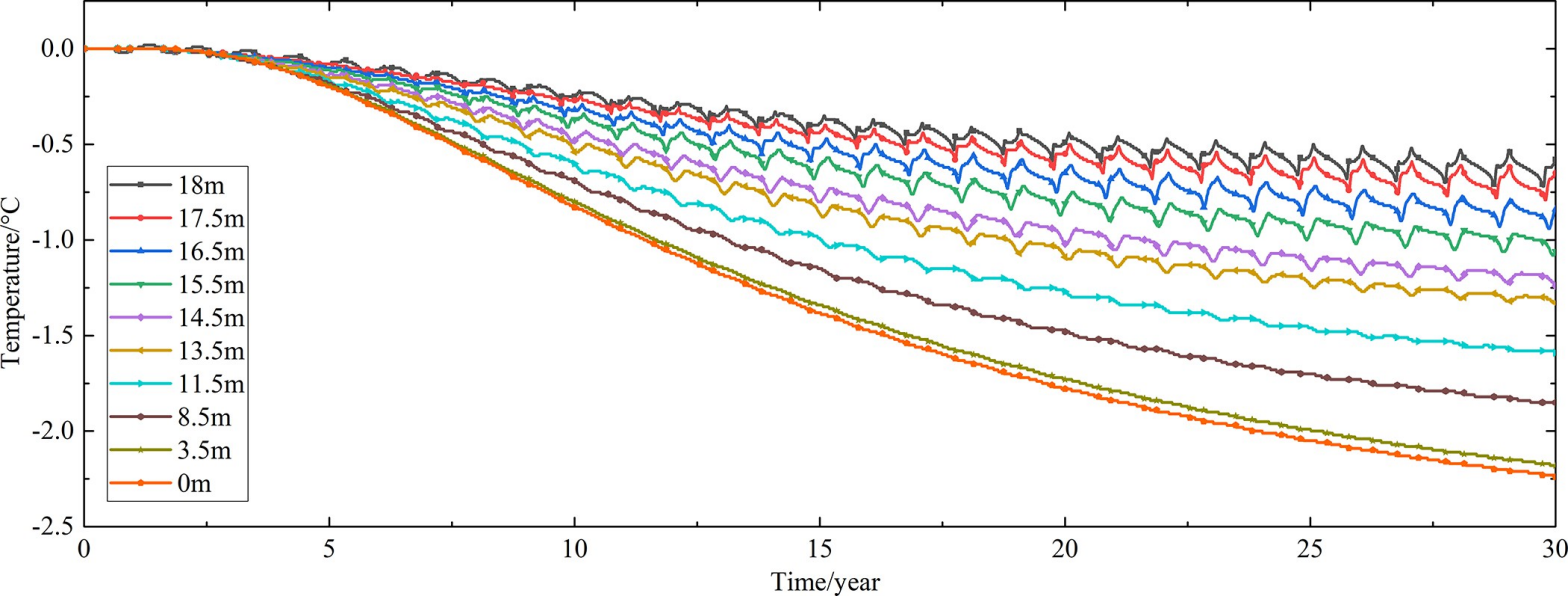
Temperature time-history curves at the depth of 0m-18m for the temperature difference between the non-ventilation subgrade and the parallel perforated ventilation subgrade in the center of the natural ground.

Temperature time-history curves of the non-ventilation subgrade at the depth of 19.25m-21.225m are shown in Fig 10. The temperature difference between the non-ventilation subgrade and the parallel perforated ventilation subgrade at the depth of 19.25m-21.225m is shown in Fig 11. Temperature time-history curves of the non-ventilation subgrade at the depth of 0m-18m are shown in Fig 12. The temperature difference between the non-ventilation subgrade and the parallel perforated ventilation subgrade at the depth of 0m-18m is shown in Fig 13.

Fig 10 shows that the temperature of the non-ventilation subgrade in the center of the natural ground changes periodically with the ambient temperature. Compared with Figs 7 and 11 shows that the temperature difference between the non-ventilation subgrade and the parallel perforated ventilation subgrade also decreases with the increasing of the distance away from the perforated ventilation. However, the cooling effect of the parallel perforated ventilation subgrade on the natural ground is little at the depth of 19.25m-21.225m. Fig 12 shows that the temperature at the depth of 0m-18m increase with the increasing of the time. Compared with Figs 9 and 13 shows that the cooling effect of the parallel perforated ventilation subgrade on the natural ground is also little at the depth of 0m-18m. Fig 13 shows that the temperature difference between the non-ventilation subgrade and the parallel perforated ventilation subgrade increases with the increasing of the time and depth. The temperature difference at the depth of 0m-18m is different when the perforated ventilation works more than three years. The temperature difference begins to increase when the perforated ventilation begins to work. The temperature difference reaches the maximum value when the perforated ventilation stops working, and then the temperature difference begins to decrease. The reason is that the temperature at the depth of 19.25m-21.225m are affected by the ambient temperature and the ambient temperature is affected by the effect of global warming. The temperature at the depth of 0m-18m is little affected by the ambient temperature. The cooling effect of the parallel perforated ventilation subgrade is stronger than the effect of global warming, and the cooling effect of the parallel perforated ventilation subgrade is stronger and stronger with the increasing of time. Moreover, because the convection heat transfer and heat conduction all occur in the crushed rock layer, the cooling effect of the parallel perforated ventilation subgrade is most significant in the crushed rock layer. In addition, energy loss occurs when temperature is transferred to depth. Thereby, the follow-up study focuses on the cooling effect of the parallel perforated ventilation subgrade in the center of pavement.

### Temperature-depth curves for the non-ventilation subgrade and the parallel perforated ventilation subgrade

**Fig 14:**
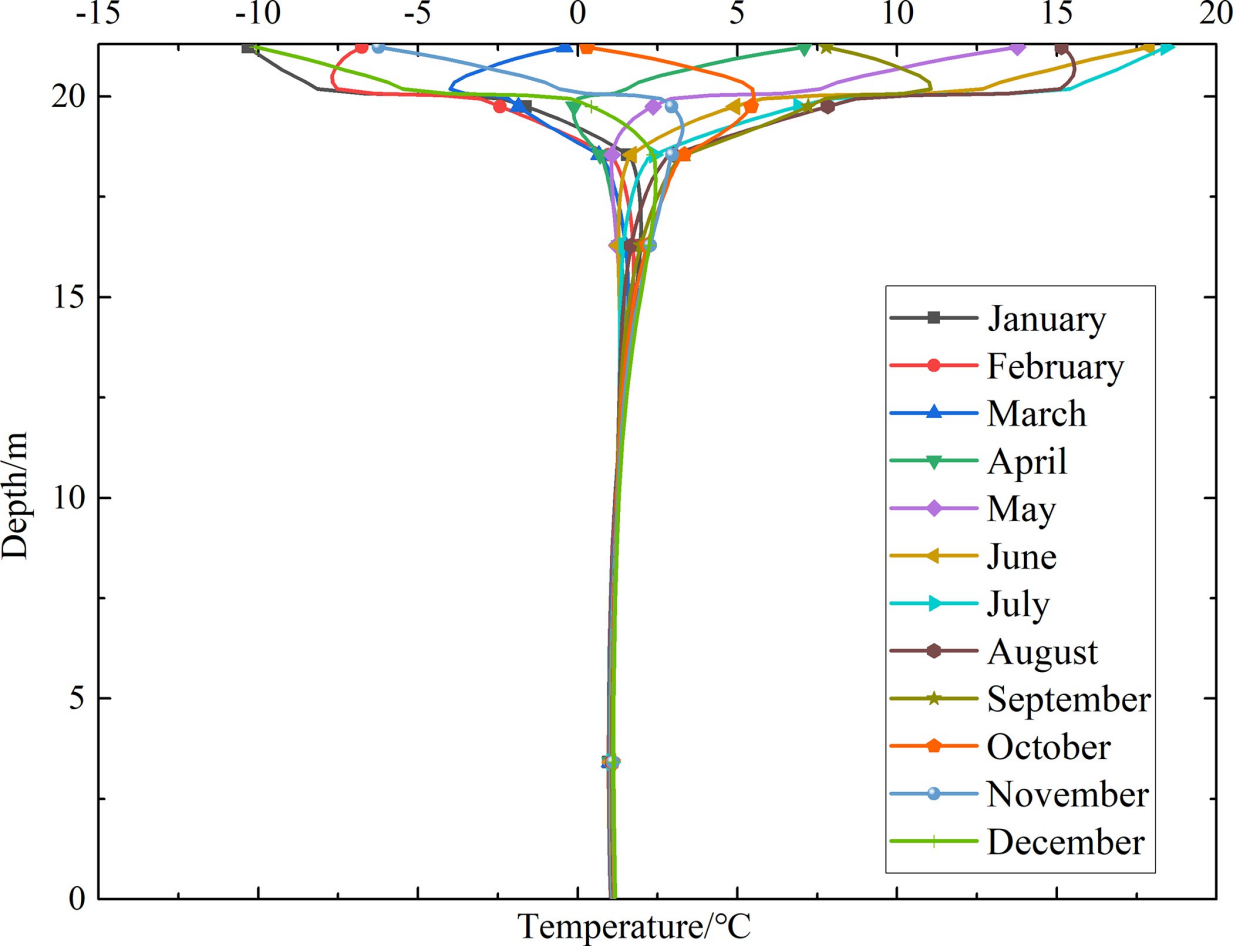
Temperature-depth curves for the non-ventilation subgrade in the tenth year.

**Fig 15:**
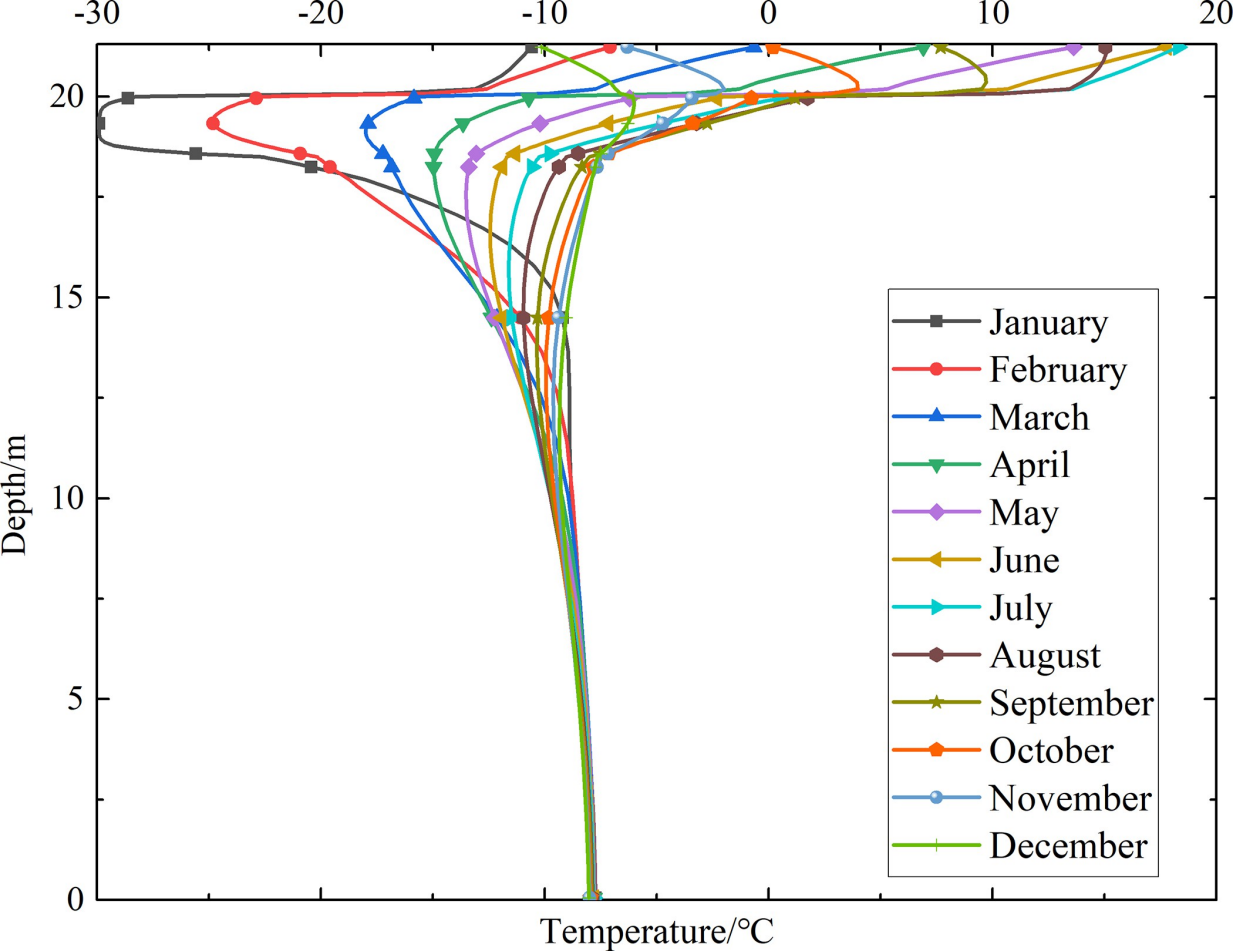
Temperature-depth curves for the parallel perforated ventilation subgrade in the tenth year.

**Fig 16:**
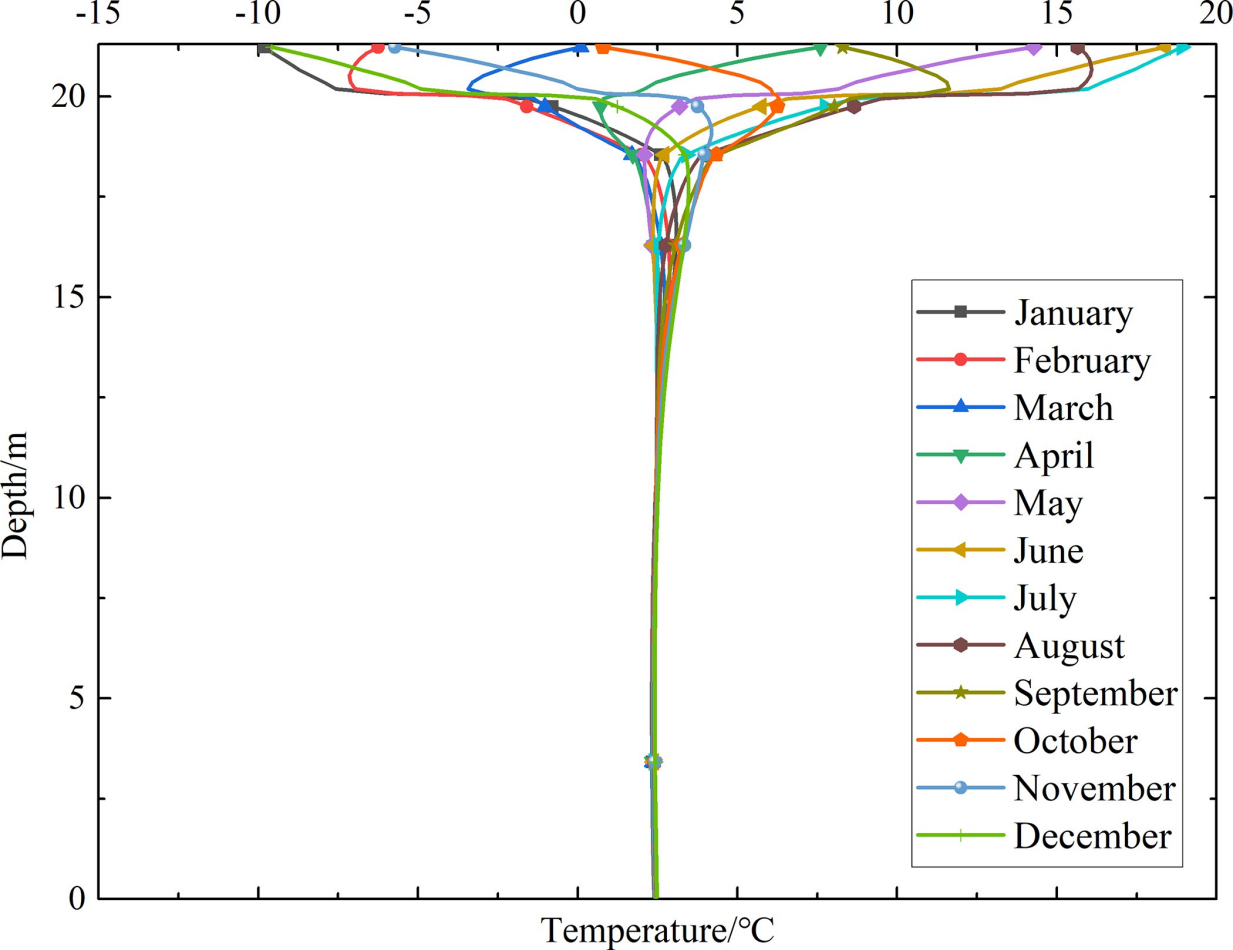
Temperature-depth curves for the non-ventilation subgrade in the twentieth year.

**Fig 17:**
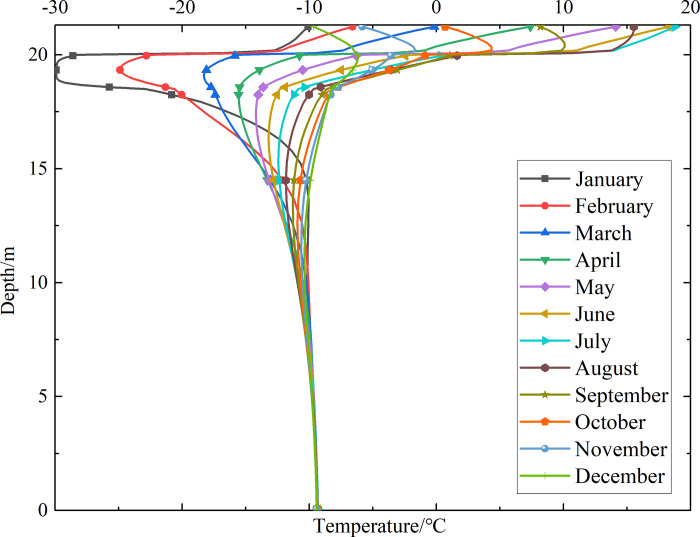
Temperature-depth curves for the parallel perforated ventilation subgrade in the twentieth year.

**Fig 18:**
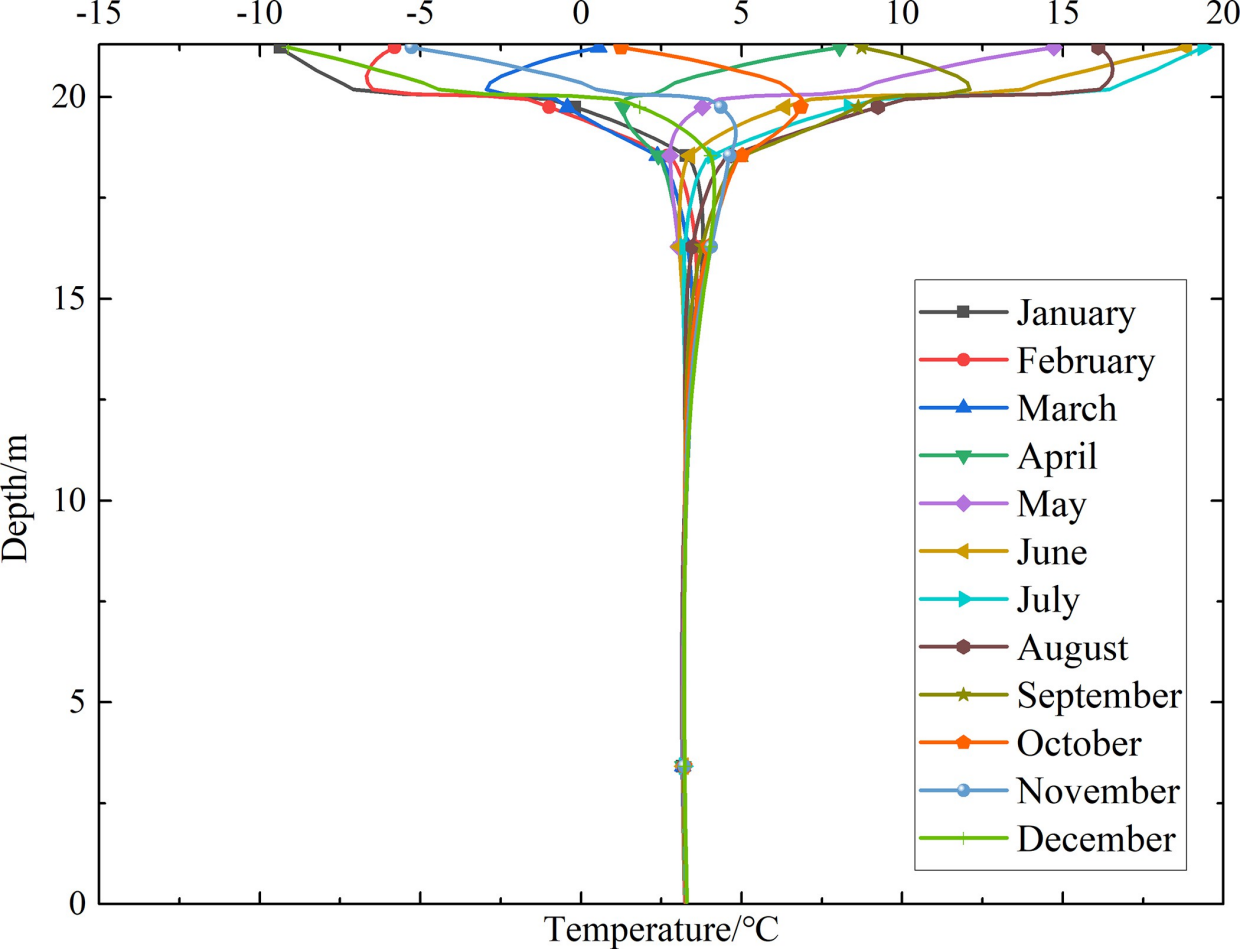
Temperature-depth curves for the non-ventilation subgrade in the thirtieth year.

**Fig 19:**
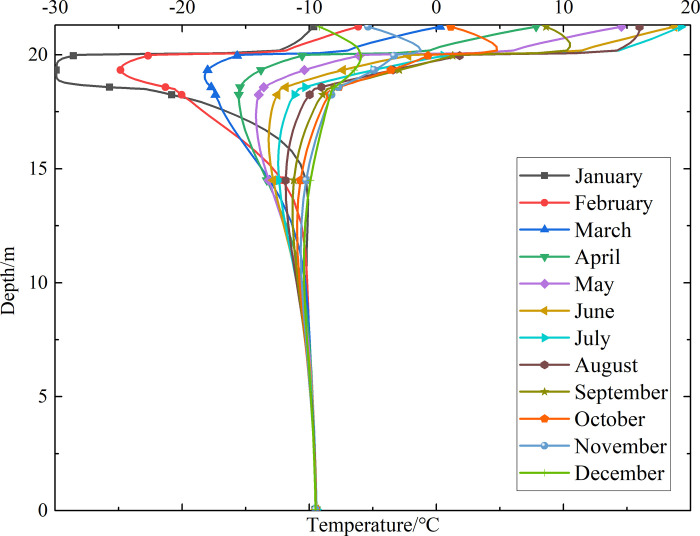
Temperature-depth curves for the parallel perforated ventilation subgrade in the thirtieth year.

To investigate the influence of month on the temperature of the non-ventilation subgrade and the parallel perforated ventilation subgrade at different depths, temperature-depth curves for the non-ventilation subgrade and the parallel perforated ventilation subgrade in the center of the pavement are shown in Figs 14–19 on the 15th day of each month for the tenth, twentieth, and thirtieth year.

Figs 14, 16 and 18 show that temperature-depth curves for the non-ventilation subgrade are roughly a funnel-shaped distribution. And the temperature tends to be stable eventually with the increase of the depth. However, the temperature changes suddenly at the insulation layer because the insulation layer prevents heat transfer. Temperature-depth curves shift to the left, which indicates the existence of the low temperature interlayer in the pavement. And temperature-depth curves shift to the right, which indicates the existence of the high temperature interlayer in the pavement. Temperature characteristics in Figs 14, 16 and 18 are associated with the study of highway and railway engineering [31,32]. Fig 14 shows that the temperature at the depth of 0m-10m tend to 1.05°C in the tenth year. Fig 16 shows that the temperature at the depth of 0m-10m tend to 2.45°C in the twentieth year. Fig 18 shows that the temperature at the depth of 0m-10m tend to 3.25°C in the thirtieth year. Hence, the stable temperature for the non-ventilation subgrade increases with the increase of time because of the effect of global warming. And the increasing amplitude of temperature decreases with the increase of time.

Figs 15, 17 and 19 show that the temperature-depth curves for the parallel perforated ventilation subgrade also change suddenly at the insulation layer, which is similar to the temperature-depth curves for the non-ventilation subgrade. The temperature for the parallel perforated ventilation subgrade fluctuates larger than that for the non-ventilation subgrade in each month at the depth of 15m-21.3m. The temperature fluctuation is most remarkable in the perforated ventilation area. The amplitude of temperature fluctuation decreases gradually in each month at the depth of 7.5m-15m, which is also larger than that for the non-ventilation subgrade. The temperature tends to a stable value at the depth of 0m-7.5m. Fig 15 shows that the temperature at the depth of 0m-10m tend to -7.85°C in the tenth year. Fig 17 shows that the temperature at the depth of 0m-10m tend to -9.35°C in the twentieth year. Fig 19 shows that the temperature at the depth of 0m-10m tend to -9.50°C in the thirtieth year. Compared with the temperature for the non-ventilation subgrade, the stable temperature for the parallel perforated ventilation subgrade decreases 8.9°C in the tenth year, 11.8°C in the twentieth year, and 12.73°C in the thirtieth year. The stable temperature for the parallel perforated ventilation subgrade decreases with the increase of time, and the decreasing amplitude of temperature decreases with the growth of time. The reason is that the insulation layer is at the upper of the crushed rock layer, and the perforated ventilation is located in the center of the crushed rock layer. The insulation layer prevents heat transfer from the pavement as well as cold transfer from the perforated ventilation. Hence, the pavement above the insulation layer is significantly affected by the ambient temperature and shows the characteristics of seasonal temperature changes. Meanwhile, the pavement and subgrade under the insulation layer are significantly affected by the perforated ventilation and show the obvious cooling effect.

### The influences of air velocity and working time on the temperature of the parallel perforated ventilation subgrade

**Fig 20:**
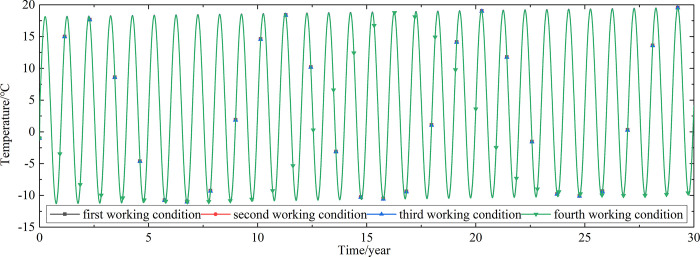
Temperature time-history curves at the depth of 21.225m.

**Fig 21:**
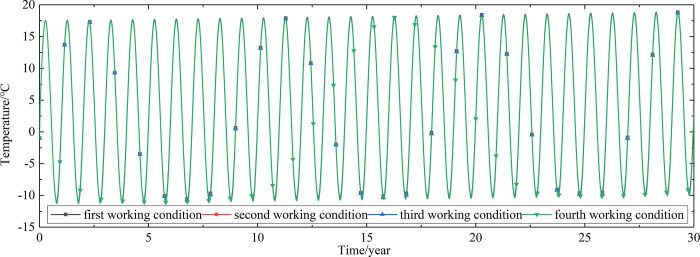
Temperature time-history curves at the depth of 21.075m.

**Fig 22:**
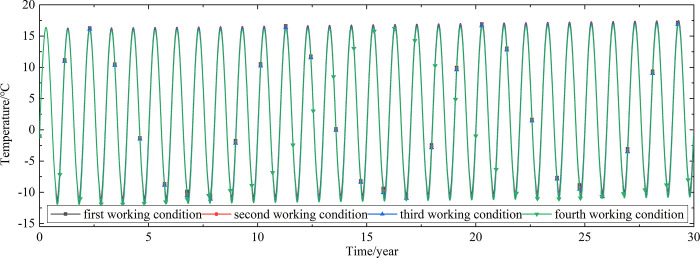
Temperature time-history curves at the depth of 20.8m.

**Fig 23:**
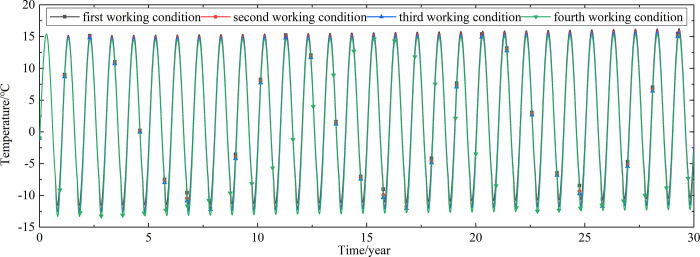
Temperature time-history curves at the depth of 20.35m.

**Fig 24:**
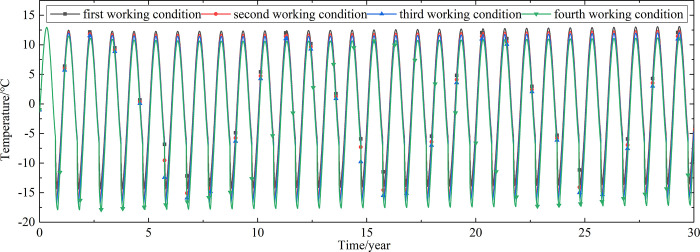
Temperature time-history curves at the depth of 20.05m.

**Fig 25:**
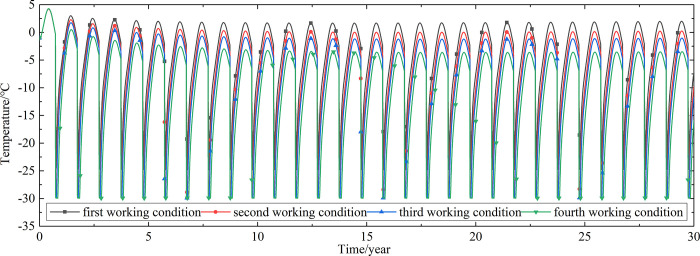
Temperature time-history curves at the depth of 19.25m.

**Fig 26:**
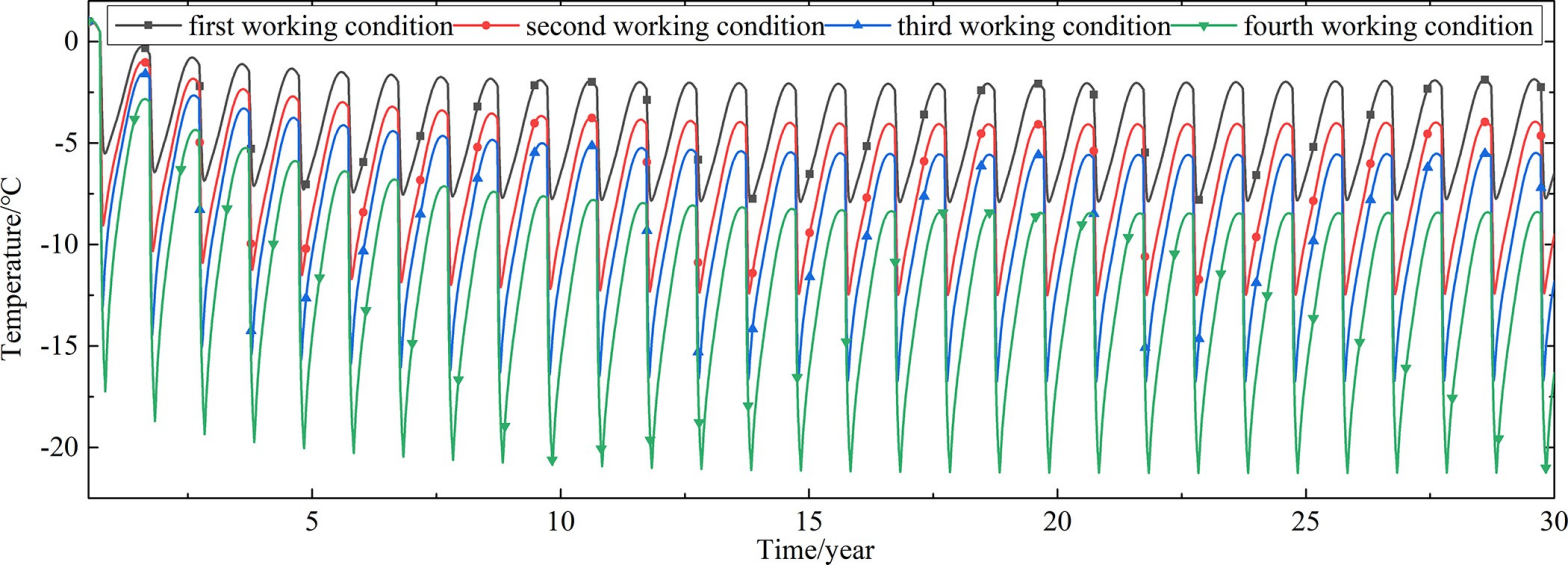
Temperature time-history curves at the depth of 18m.

**Fig 27:**
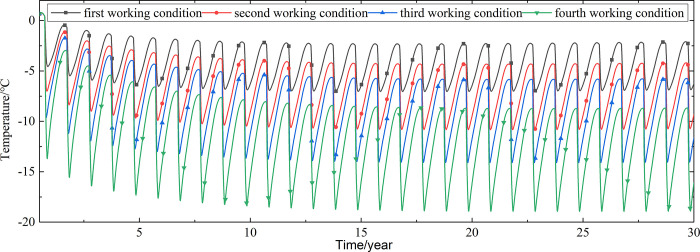
Temperature time-history curves at the depth of 17.5m.

**Fig 28:**
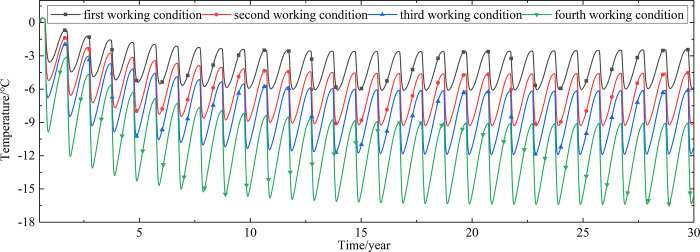
Temperature time-history curves at the depth of 16.5m.

**Fig 29:**
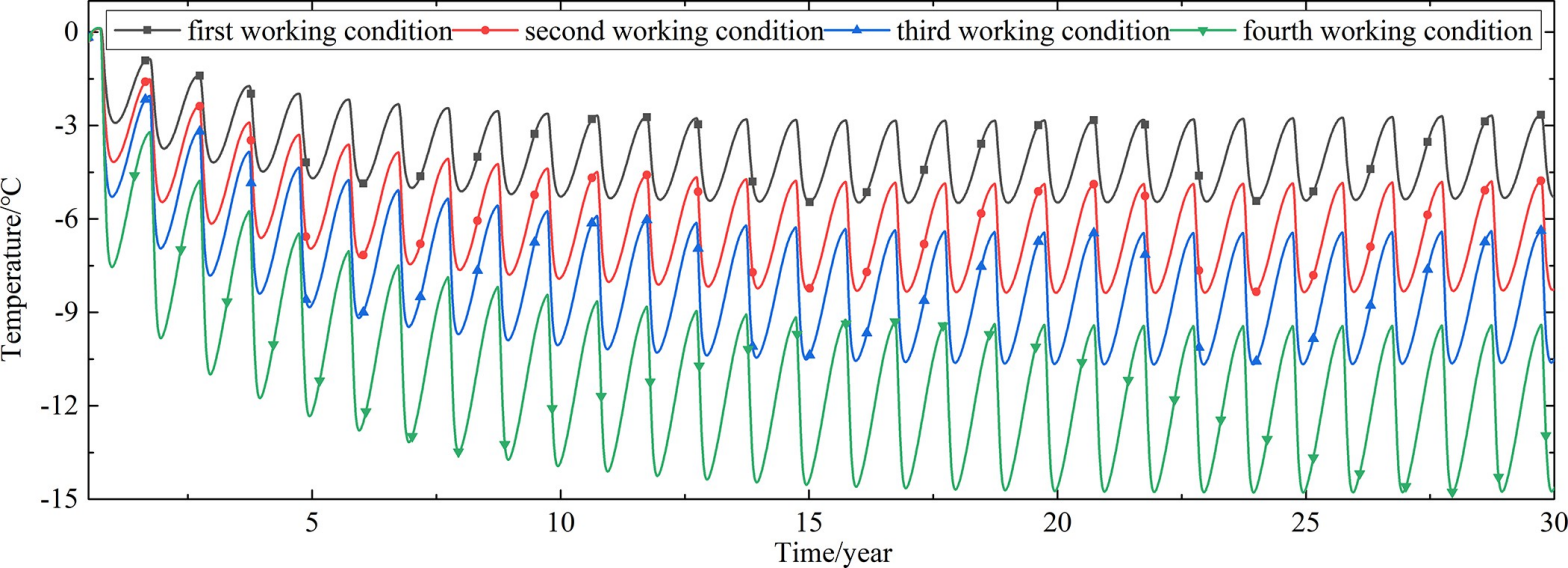
Temperature time-history curves at the depth of 15.5m.

**Fig 30:**
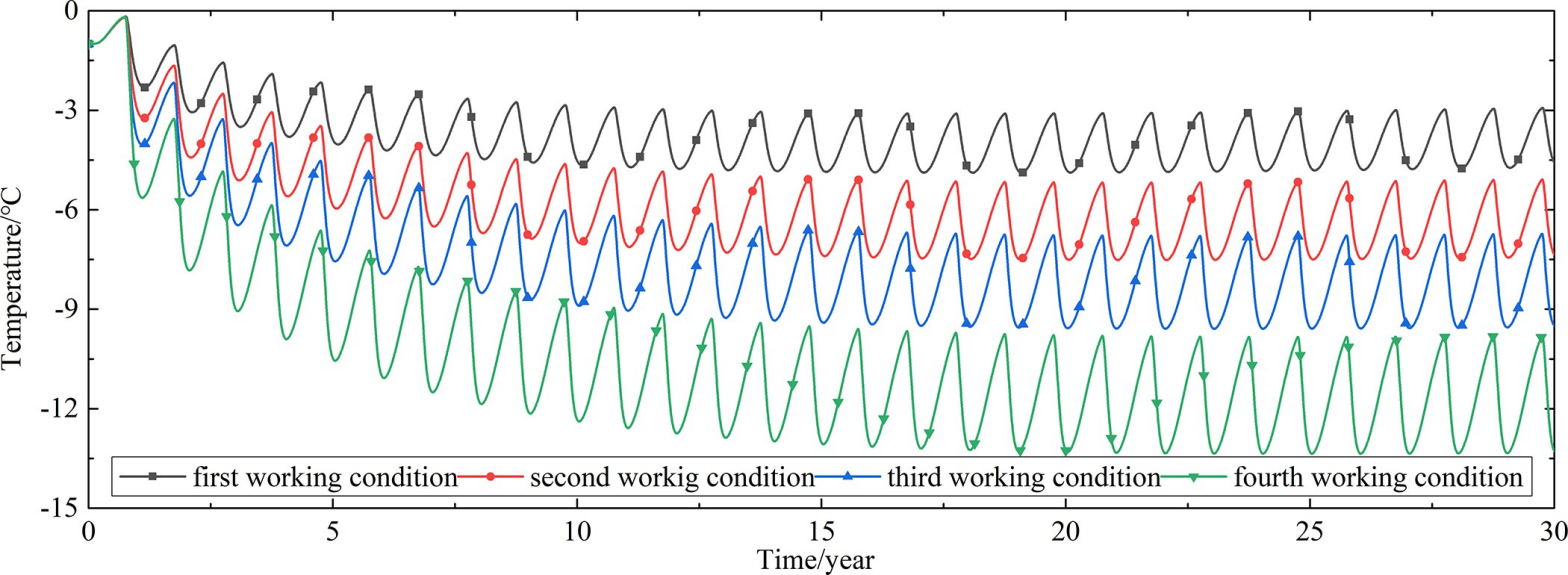
Temperature time-history curves at the depth of 14.5m.

**Fig 31:**
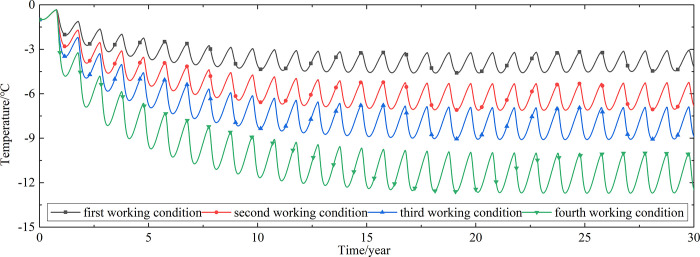
Temperature time-history curves at the depth of 13.5m.

**Fig 32:**
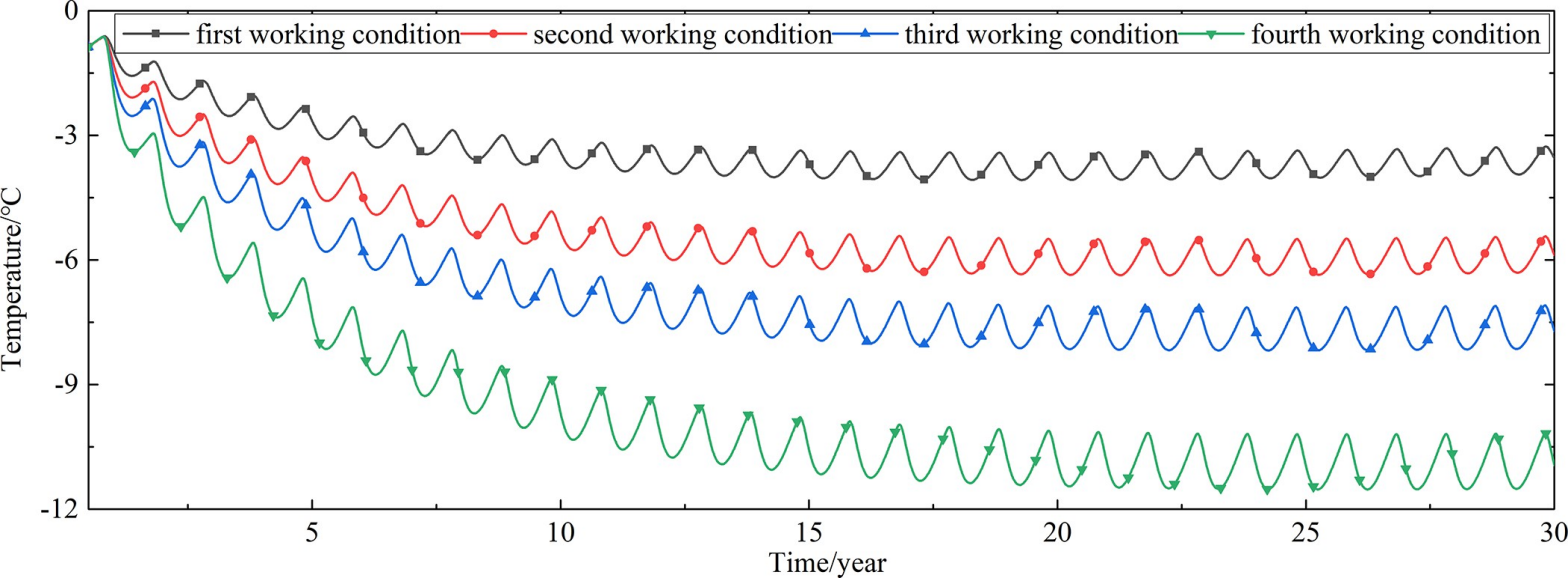
Temperature time-history curves at the depth of 11.5m.

**Fig 33:**
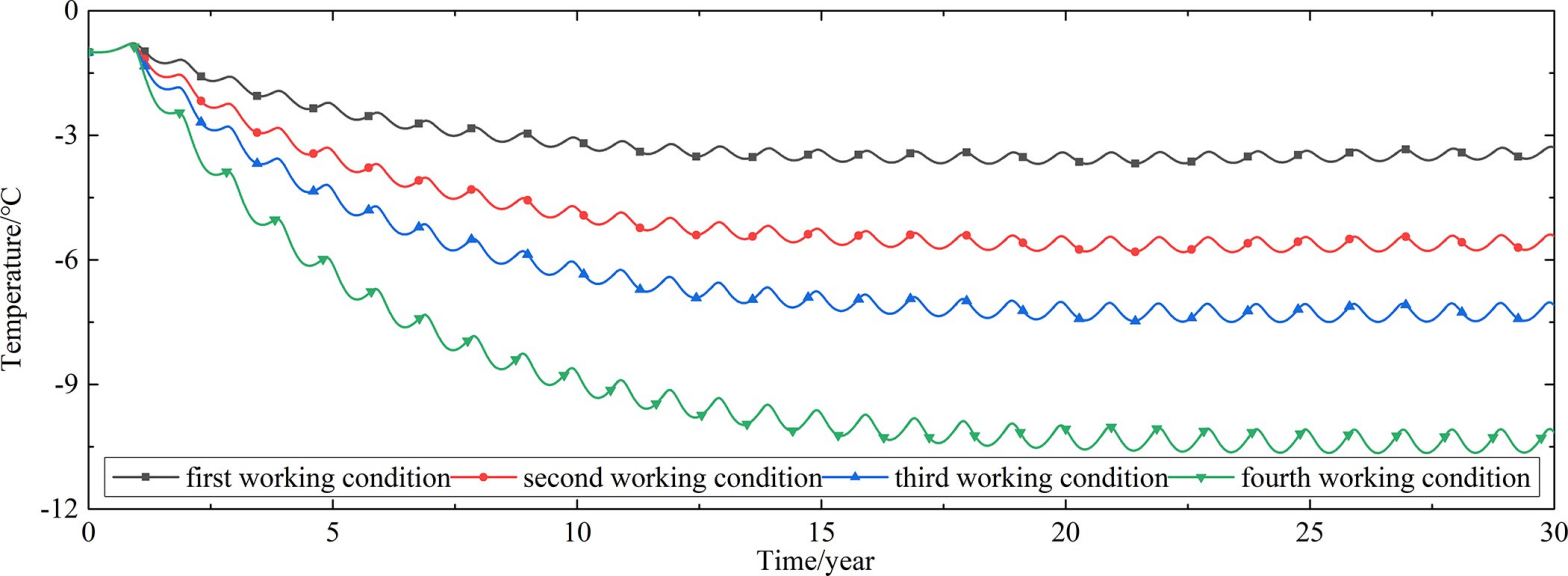
Temperature time-history curves at the depth of 8.5m.

**Fig 34:**
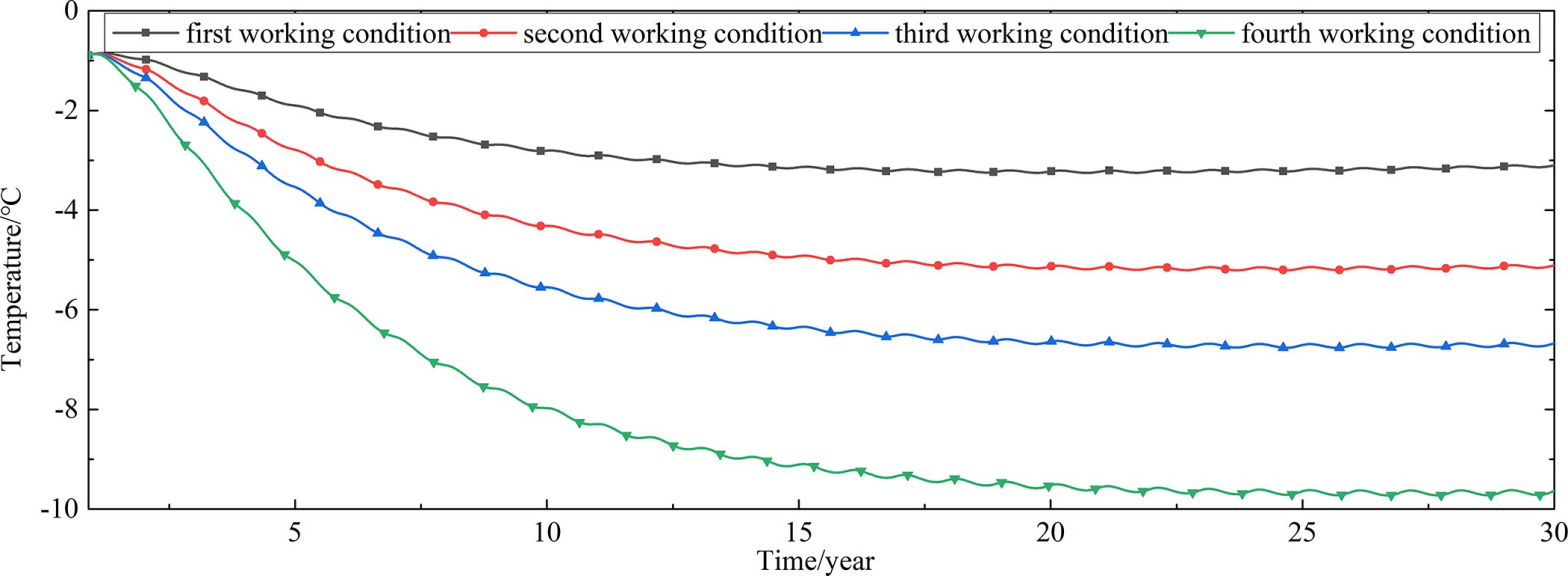
Temperature time-history curves at the depth of 3.5m.

**Fig 35:**
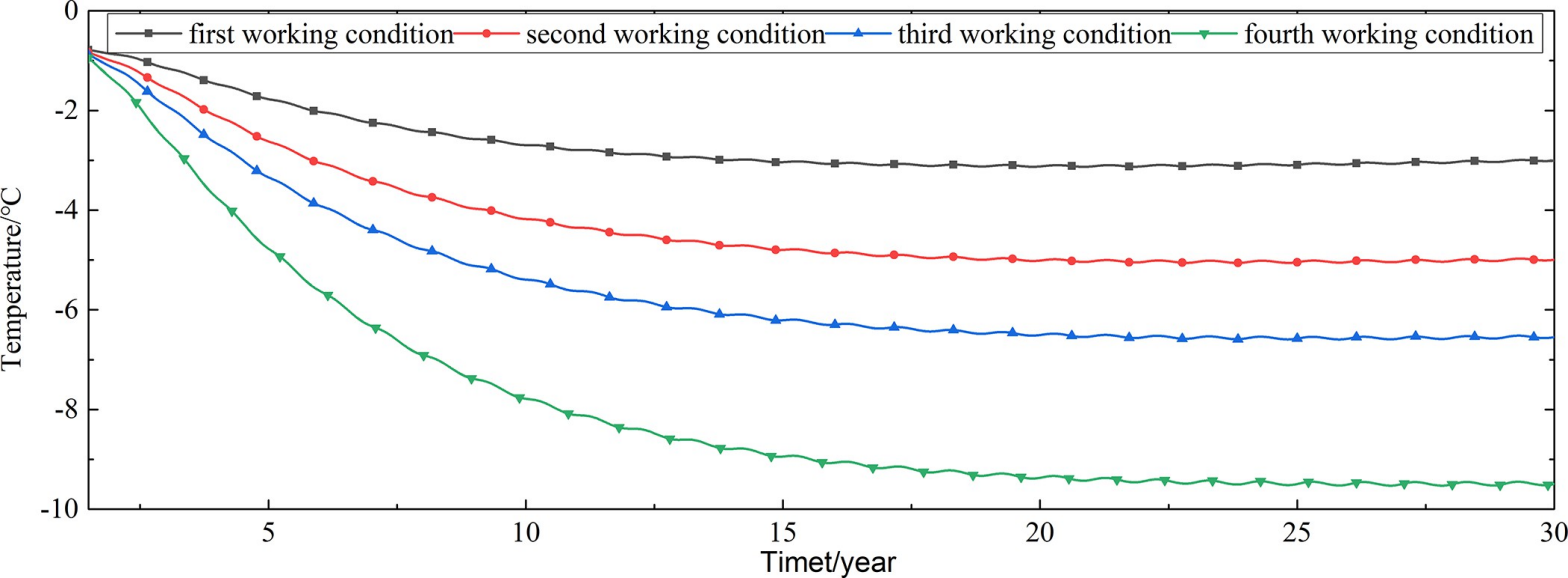
Temperature time-history curves at the depth of 0m.

To explore the influences of air velocity and working time on the cooling effect of the parallel perforated ventilation subgrade in the center of the pavement, temperature time-history curves at different air velocities and working time are shown in Figs 20–35. The first working condition is the air velocity of 1m/s and the working time of 20 days. The second working condition is the air velocity of 2m/s and the working time of 20 days. The third working condition is the air velocity of 5m/s and the working time of 20 days. The fourth working condition is the air velocity of 5m/s and the working time of 40 days. The working period of 20 days for the parallel perforated ventilation is from December 22 to January 10 every year.

Figs 20–23 show that the temperature of the surface layer, base layer, and subbase layer change periodically every year, and increase slowly with the growth of time. And the temperature of the surface layer, base layer, and subbase layer have no change at different working conditions. Fig 24 shows that the temperature of the insulation layer changes periodically every year and increases slowly with the growth of time. And the temperature of the insulation layer has little change in different working conditions. Fig 25 shows that the temperature of the crushed rock layer decreases when it is from the first working condition to fourth working condition, which means that the temperature of the crushed rock layer decreases with the growth of the air velocity and working time. When the time is more than twenty years, the temperature of the crushed rock layer keeps in a stable range. When the time is from the twentieth year to the thirtieth year, the temperature fluctuation of the crushed rock layer is -24.8°C~2°C for the first working condition, -29.4°C~0.3°C for the second working condition, -29.9°C~-1°C for the third working condition, and -29.9°C~-3.5°C for the fourth working condition. Figs 26–33 show that the temperature of the subgrade changes periodically every year and is negative for thirty years. When the time is more than twenty years, the temperature of the subgrade keeps in a stable range and decreases with the growth of the depth. Figs 34 and 35 show that the temperature of the subgrade decreases with the growth of time and tends to a stable range when the time is more than twenty years. When it is from the first working condition to the fourth working condition, the temperature of the subgrade decreases with the increasing of the air velocity and working time. The reason is that the surface layer, base layer, and subbase layer are very close to the ambient environment and are significantly affected by the environmental temperature. And the environmental temperature is affected by the effect of global warming. However, the insulation layer is far away from the ambient environment and prevents the temperature from transferring. The perforated ventilation is in the crushed rock layer, and the cold energy from the perforated ventilation decreases the temperature of the crushed rock layer by heat convection and heat conduction. The cold energy from the perforated ventilation dissipates gradually in the process of temperature transfer along the depth.

## Conclusions

As a new type of ventilation, the parallel perforated ventilation is proposed to maintain the temperature stability of the subgrade of airport engineering in the permafrost region. And the applicability and reliability of the finite element model of the runway with the parallel perforated ventilation subgrade are verified by comparing with the previous studies. Moreover, the cooling effect of the parallel perforated ventilation subgrade in airport engineering is analyzed by comparing temperature time-history curves and temperature-depth curves for the non-ventilation subgrade and the parallel perforated ventilation subgrade. And the influence of the air velocity and working time on the cooling effect of the parallel perforated ventilation subgrade in airport engineering are also explored. The conclusions are as follows:

1. For the non-ventilation subgrade in airport engineering, the temperature of the surface layer, base layer, subbase layer, insulation layer, crushed rock layer, subgrade and natural ground all change periodically with the ambient temperature each year and increase with the growth of time. The influence of the ambient temperature at the depth of 20.8m-21.225m is most significant for the pavement and the natural ground. The influence of the ambient temperature at the depth of 19.25m-20.35m is less significant for the pavement and the natural ground. The influence of the ambient temperature at the depth of 0m-18m is little significant for the pavement and the natural ground. In the center of the pavement, the temperature at the depth of Y = 18m, Y = 17.5m, Y = 16.5m, Y = 15.5m, Y = 14.5m, and Y = 13.5m are negative only in the winter of the first two years and are positive the whole year for the rest of the time. The temperature at the depth of Y = 11.5m is negative only for the first four years and is positive the whole year for the rest of the time. And the temperature at the depth of Y = 8.5m, Y = 3.5m, and Y = 0m are negative only for the first five years and are positive the whole year for the rest of the time.
2. The cooling effect of the parallel perforated ventilation subgrade in airport engineering is stronger than the effect of global warming. The temperature of the surface layer, base layer, subbase layer, insulation layer, crushed rock layer, subgrade and natural ground for the parallel perforated ventilation subgrade are less than those for the non-ventilation subgrade in airport engineering. Compared with the non-ventilation subgrade in airport engineering, the lowest temperature is in the crushed rock layer for thirty years. The crushed rock layer and subgrade are positive temperature only in spring and summer of the first year and are negative temperature in the whole year for the rest of the time. The temperature difference between the non-ventilation subgrade and the parallel perforated ventilation subgrade in airport engineering decreases with the increasing of the distance away from the perforated ventilation. The temperature difference begins to increase when the perforated ventilation begins to work. The temperature difference reaches the maximum value when the perforated ventilation stops working, and then the temperature difference begins to decrease. For the parallel perforated ventilation subgrade in airport engineering, the temperature at the depth of Y = 18m, Y = 17.5m, Y = 16.5m, Y = 15.5m, Y = 14.5m, Y = 13.5m and Y = 11.5m are positive only in the summer of the first year and are negative the whole year for the rest of the time. The temperature at the depth of Y = 8.5m, Y = 3.5m, and Y = 0m are negative in the whole year for the thirty years. The cooling effect of the parallel perforated ventilation subgrade in the center of the pavement is stronger than that in the center of the natural ground. The temperature difference between the non-ventilation subgrade and the parallel perforated ventilation subgrade increases with the increasing of the time and depth. The temperature difference at the depth of 0m-18m is different when the perforated ventilation in airport engineering works more than three years.
3. Temperature-depth curves for the non-ventilation subgrade and the parallel perforated ventilation subgrade all change suddenly at the insulation layer in airport engineering. Temperature-depth curves shift to the left, indicating the existence of low temperature interlayers in the pavement. And temperature-depth curves shift to the right, indicating the existence of high temperature interlayers in the pavement. The stable temperature for the non-ventilation subgrade in airport engineering increases with the increase of time, and the increasing amplitude of temperature decreases with the growth of time. The stable temperature for the parallel perforated ventilation subgrade in airport engineering decreases with the increase of time, and the decreasing amplitude of temperature decreases with the growth of time. The temperature for the parallel perforated ventilation subgrade in airport engineering fluctuates larger than that for the non-ventilation subgrade in each month at the depth of 15m-21.3m. The temperature fluctuation is most remarkable in the perforated ventilation. The temperature fluctuation amplitude decreases gradually each month at the depth of 7.5m-15m, which is also larger than that for the non-ventilation subgrade in airport engineering. The temperature tends to be a stable value at the depth of 0m-7.5m for the parallel perforated ventilation subgrade in airport engineering.
4. The air velocity and working time of the parallel perforated ventilation in airport engineering have no effect on the temperature of the surface layer, base layer, and subbase layer, have little effect on the temperature of the insulation layer, and have great effect on the temperature of the crushed rock layer and subgrade. For the parallel perforated ventilation subgrade in airport engineering, as the air velocity and working time increase, the temperature of the crushed rock layer and subgrade keep in a stable range when the time is more than twenty years. The temperature of the crushed rock layer and subgrade at the depth of 8.5m-18m change periodically every year, but the temperature of the subgrade at the depth of 0m-3.5m decreases in nonlinear for the first twenty years.
